# Functionalization and Antibacterial Applications of Cellulose-Based Composite Hydrogels

**DOI:** 10.3390/polym14040769

**Published:** 2022-02-16

**Authors:** Yunhui Bao, Jian He, Ke Song, Jie Guo, Xianwu Zhou, Shima Liu

**Affiliations:** 1 Key Laboratory of Hunan Forest Products and Chemical Industry Engineering, Jishou University, Zhangjiajie 427000, China; baoyunhui1999@foxmail.com (Y.B.); jianhelchg@jsu.edu.cn (J.H.); kesong@jsu.edu.cn (K.S.); guojie1981@jsu.edu.cn (J.G.); zhouxianwu@jsu.edu.cn (X.Z.); 2 College of Chemistry and Chemical Engineering, Jishou University, Jishou 416000, China

**Keywords:** cellulose, hydrogels, antibacterial, biomedical, nanotechnology

## Abstract

Pathogens, especially drug-resistant pathogens caused by the abuse of antibiotics, have become a major threat to human health and public health safety. The exploitation and application of new antibacterial agents is extremely urgent. As a natural biopolymer, cellulose has recently attracted much attention due to its excellent hydrophilicity, economy, biocompatibility, and biodegradability. In particular, the preparation of cellulose-based hydrogels with excellent structure and properties from cellulose and its derivatives has received increasing attention thanks to the existence of abundant hydrophilic functional groups (such as hydroxyl, carboxy, and aldehyde groups) within cellulose and its derivatives. The cellulose-based hydrogels have broad application prospects in antibacterial-related biomedical fields. The latest advances of preparation and antibacterial application of cellulose-based hydrogels has been reviewed, with a focus on the antibacterial applications of composite hydrogels formed from cellulose and metal nanoparticles; metal oxide nanoparticles; antibiotics; polymers; and plant extracts. In addition, the antibacterial mechanism and antibacterial characteristics of different cellulose-based antibacterial hydrogels were also summarized. Furthermore, the prospects and challenges of cellulose-based antibacterial hydrogels in biomedical applications were also discussed.

## 1. Introduction

At present, the threat of bacterial contamination has attracted much attention in the drinking water [1], food [2], and medical and health industries [3,4]. In particular, diseases and deaths caused by pathogen infection pose a great challenge to human health and public health safety, resulting in the loss of nearly 10 million lives worldwide every year. From the time penicillin was discovered to the present, antibiotics have held a significant position in fighting multiple bacterial infections while contributing to the defense of human health [5,6]. By contrast, tremendous abuse and misuse of antibiotics have led to the subsequent emergence of many drug-resistant bacteria, such as fidaxomicin-resistant *Enterococci* (K-1476) and methicillin-resistant *Staphylococcus aureus* (*S. aureus*) [7]. However, the drug resistance of bacteria caused by the abuse of antibiotics has been listed as one of the greatest health threats faced by human beings by the World Health Organization (WHO) [8,9]. The emergence of multiple drug-resistant bacteria further limits the application of existing antibacterial agents. As a consequence, it is necessary to develop new antibacterial materials at the two levels of basic theoretical research and practical application.

Hydrogels, a three-dimensional network formed by cross-linking of hydrophilic polymers through physical or chemical bonds, can effectively absorb and lock a large number of water molecules while maintaining its mechanical and physical morphology (owing to its cyberspace) [10]. The three-dimensional network structure of hydrogel endows hydrogel with superior adsorption and release properties. On the one hand, besides absorbing water molecules, it will also adsorb certain impurities in water during the swelling process of hydrogel. Hence, hydrogel is usually applied to remove heavy metal ions from water [11]. Then again, the perfect biocompatibility of hydrogel makes it potential to be utilized in biomedicine, et al. [12]. In addition, the porous structure of hydrogels makes them have a nicely controlled release ability for most antibacterial agents. Therefore, hydrogels have turned into very appropriate materials for carrying antibacterial agents. Meanwhile, it has been broadly used to load related antibacterial drugs. Thus, the growth of antibacterial composite hydrogels has become a popular topic [13,14].

Cellulose, one of the most abundant natural biopolymers available in nature, is a natural polymer formed by D-type glucose unit polymerization, which can be extracted from plants or a small number of bacteria (such as *Acetobacter xylinum*) [15,16,17]. Cellulose has been widely adopted in the pharmaceutical, medical, papermaking, textile, and other fields owing to its many attractive advantages such as biodegradability, excellent biocompatibility, non-toxicity, low cost, and good thermal/chemical stability [18,19,20]. Along with this, the widespread application of cellulose has also resulted in notable economic benefits. The market size of cellulose was nearly 146.7 million dollars in 2019 according to the global market research. Furthermore, it is predicted that the market size of cellulose will grow at a compound annual growth rate of 21.4% between 2020 and 2026 [21].

**Figure 1:**
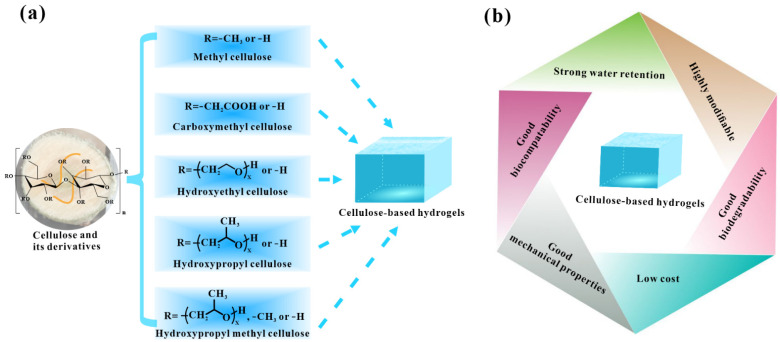
Representative cellulose derivatives for synthesis of cellulose-based hydrogels (**a**) and advantages of cellulose-based hydrogels in practical applications (**b**).

For cellulose or its derivatives, it is easy to modify and cross-link with one another since cellulose (or its derivatives) is a long-chain linear polymer and contains rich hydrophilic functional groups, including hydroxyl, carboxyl, and aldehyde groups [22], which make cellulose or its derivatives to be an ideal raw material for the synthesis of hydrogels (Figure 1a). To date, in addition to cellulose, several cellulose derivatives have been created and adopted to prepare cellulose-based hydrogels. Moreover, the properties of these cellulose derivatives are different, and each one has its advantages and disadvantages, respectively (Table 1). For example, hydroxypropyl cellulose tends to cause allergic reactions in humans or animals during its use [23]. However, hydroxypropyl cellulose has better chemical stability and water solubility than other common cellulose derivatives [24,25].

Cellulose-based hydrogels have the advantages of strong water retention, good biodegradability, good biocompatibility, highly modifiable, low cost, and good mechanical properties (Figure 1b). As a consequence, cellulose-based hydrogels are widely adopted in tissue engineering [50], controllable delivery systems [51], blood purification [52], sensor [53], agriculture [54], water purification [55], and chromatographic carriers [56]. Cellulose-based hydrogels, composed of non-toxic and biocompatible cellulose and water molecules, does not show any pristine antibacterial activity. However, cellulose has excellent modifiability and the ability to combine with antibacterial materials, which makes cellulose-based hydrogels have great potential to develop into antibacterial hydrogels [57,58]. In the process of integrating cellulose hydrogels with antibacterial materials, the properties (e.g., swelling and adsorption) of the corresponding antibacterial hydrogels can be improved by modifying the variety and addition number of antibacterial agents [59]. Current studies have shown that cellulose-based antibacterial hydrogels are a very excellent antibacterial material and have a broad application prospect in the field of biomedicine [60,61].

Although there have been some reviews on the preparation, properties, and applications of cellulose-based hydrogels, Reviews concerning the antibacterial applications of cellulose hydrogels are few. As a consequence, the antibacterial properties and applications of a variety of cellulose-based hydrogels, including cellulose and its derivatives with metal nanoparticles, metal oxide nanoparticles, antibiotics, polymers or plant extracts to form cellulose-based hydrogels are systematically summarized in this contribution. Meanwhile, the preparation methods of cellulose-based composite hydrogels are also introduced. It is expected to provide theoretical basis and new ideas for the design and application of cellulose-based and other biomass-based antibacterial materials in the future.

## 2. Preparation of Cellulose-Based Composite Hydrogels

Since hydrogel is a complex formed from the combination of three-dimensional hydrophilic polymer network and water molecules, the key to prepare cellulose-based hydrogels is to prepare a cellulose-based three-dimensional network. Crosslinking is the core step in the preparation of three-dimensional network of cellulose [62,63]. The existing cellulose crosslinking technology can be generally divided into two categories: physical crosslinking and chemical crosslinking. The corresponding hydrogels are called chemical hydrogels and physical hydrogels respectively [64]. Among them, physical crosslinking has the advantages of simple operation, no pollution, and the synthetic cellulose-based hydrogels are green and non-toxic. The preparation of hydrogels by chemical crosslinking has advantages such as high strength, a controllable structure, and adjustable properties [65].

### 2.1. Physical Crosslinking

Physical crosslinking of cellulose and its derivatives is to crosslink cellulose together through reversible physical interaction (non-covalent bond), such as entanglement, van der Waals force, ion interaction, and hydrogen bonds [66,67]. Common physical crosslinking methods include freeze-thaw technology, photoinitiation technology, and irradiation technology. Among them, freezing and thawing technology refers to the dissolution of cellulose and its derivatives in some special solvent systems, and then the cellulose-based hydrogels can be prepared by circulating freezing and thawing [67]. Wang et al. [68] mixed polyvinyl alcohol (PVA) and carboxymethyl cellulose aqueous solution in different proportions, frozen at 20 °C for 6 h, and then thawed at room temperature for 1 h. PVA-carboxymethyl cellulose composite hydrogel was prepared by repeated freezing and thawing cycles five times. Cellulose-based hydrogels prepared by photoinitiation technology have the advantages of mild reaction conditions, less by-products, and excellent biocompatibility of hydrogels. Yang et al. directly mixed the cellulose nanocrystals, polyethylene glycol (PEG) and 2-hydroxyl-1-[4-(2-hydroxyethoxy) phenyl]-2-methyl-1-propanone aqueous suspension. After degassing, the composite nanocomposite hydrogel with stronger mechanical toughness and elasticity was prepared by ultraviolet irradiation (20 W, 365 nm, 20 min) at room temperature [69]. Radiation crosslinking technology is an environmentally friendly preparation method of hydrogels [64]. Without any catalyst or crosslinking agent, hydrogels can be synthesized rapidly. Ibrahim et al. designed a acrylamide-carboxymethyl cellulose hydrogel by radiation crosslinking acrylamide monomer and carboxymethyl cellulose [70].

### 2.2. Chemical Crosslinking

**Figure 2:**
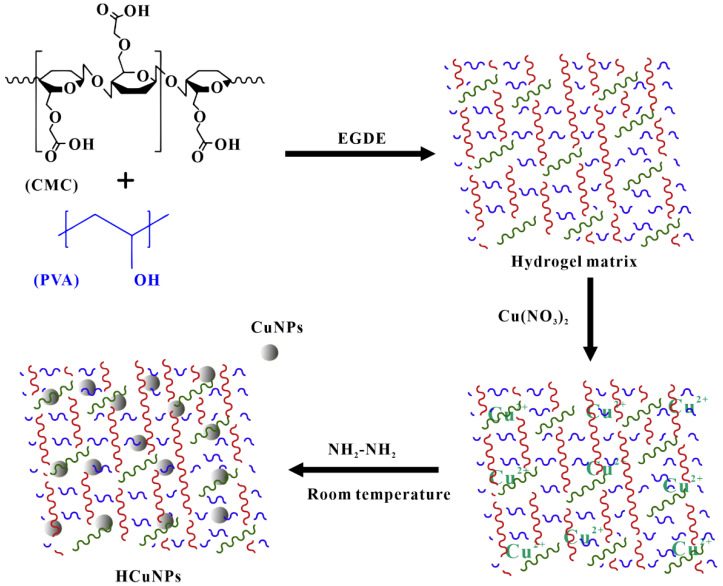
Schematic diagram of the preparation of CuNPs-cellulose hydrogel (reprinted from reference [78] with permission from Elsevier).

The preparation of cellulose-based hydrogels by chemical crosslinking usually requires the use of specific crosslinking agents to form covalent bonds in cellulose and its derivatives. The common cellulose derivatives include methyl cellulose, carboxymethyl cellulose, hydroxyethyl cellulose, hydroxypropyl cellulose and hydroxypropyl methyl cellulose (Table 1). The common crosslinking agents include citric acid, epichlorohydrin, polyethylene glycol diacrylate and glutaraldehyde (EGDE) [71,72,73]. As the covalent bond is stronger than van der Waals force, ion interaction, and hydrogen bond, cellulose-based hydrogels prepared by chemical crosslinking often possess better mechanical properties (mechanical strength and toughness) as compared to the counterpart prepared by physical methods [74]. Demitri et al. [75] reported the preparation of hydroxyethyl cellulose-carboxymethyl cellulose hydrogel with citric acid as crosslinking agent. Citric acid has the advantages of non-toxic and low cost compared with other crosslinking agents. At the same time, it was found that the swelling rate of hydrogels depends on the reaction time and the concentration of citric acid. When the concentration of citric acid is 3.75%, the swelling rate of the hydrogel can reach 900. Yan et al. [76] utilize propylene oxide crosslinked hydroxypropyl cellulose to prepare hydroxypropyl cellulose hydrogels. Marsano et al. [77] also synthesized hydroxypropyl cellulose hydrogels with hydroxypropyl cellulose as raw material and polyethylene glycol two glycidyl ether as crosslinking agent. Similarly, Al-Enizi et al. [78] adopted carboxymethyl cellulose and PVA as raw materials, EGDE as a cross-linking agent, and hydrazine hydrate as a reducing agent to prepare PVA−cellulose hydrogels containing copper nanoparticles (Figure 2). In addition to the above methods, there are some novel technologies, such as hydrogel foaming, enzyme bonding and solid-liquid interface contact method, have been developed for the preparation of unique cellulose-based hydrogels [79].

Although there are many methods for preparing cellulose-based hydrogels, every method almost has its drawbacks. Therefore, in subsequent research, we need to continue to explore better methods for preparing cellulose-based hydrogels. For example, developing methods for the simultaneous use of physical crosslinking and chemical crosslinking, cellulose-based hydrogels with two advantages of synthetic methods can be prepared. Developing a more environmentally friendly and low-cost cellulose dissolution system is necessary since the existing cellulose dissolution systems have problems including their non-environmental protection and high cost. Developing more efficient and green chemical crosslinking agents is desirable since the existing chemical cross-linking agents all have one or more disadvantages including low cross-linking efficiency, unfriendly environment, high biological toxicity, and high price.

## 3. Antibacterial Properties of Diverse Cellulose-Based Hydrogels

### 3.1. Cellulose-Based Antibacterial Hydrogels Loaded with Metal Nanoparticles

The abuse and misuse of antibiotics have led to the emergence of a large number of drug-resistant bacteria in recent decades, which has introduced severe challenges into the medical and health field [80]. As a consequence, it is urgent to develop new substitutes for antibiotics. The development of nanoscience and nanotechnology provides many new ideas and methods for the development of new antibacterial materials. It has become a new trend to use metal nanoparticles, such as silver nanoparticles (AgNPs) and copper nanoparticles (CuNPs), as antibacterial materials [81,82,83,84,85]. Metal nanoparticles have many antibacterial properties including ultra-high specific surface area, positive surface charge, and being easy to combine with negatively charged microorganisms to inhibit their growth [86,87,88,89,90]. However, metal nanoparticles are easy to agglomerate, dissociate, and oxidize, and these defects greatly limit their antibacterial applications [91,92]. As a result, it is necessary to combine metal nanoparticles with other materials to prevent their agglomeration, dissociation, and oxidation. Cellulose-based hydrogels happen to be an excellent carrier for carrying metal nanoparticles [78]. Since their incredible modifiability and slow-release, cellulose hydrogels are appropriate platforms for metal nanoparticle carriage. Metal nanoparticles can be helpfully embellished into cellulose hydrogels and then can be slow release. Notwithstanding the slow release of metal nanoparticles themselves, metal ions will also be gradually released, which will bring a lasting antibacterial result to the hydrogel [14,57].

Among the common metal nanoparticles combined with cellulose-based hydrogels, AgNPs are widely adopted (Table 2) due to their excellent broad-spectrum antibacterial properties [93], easy synthesis, and good biocompatibility [94]. Lin et al. [95] modified tannic acid (TA) and AgNPs onto the surface of cellulose nanocrystals and combined with PVA to prepare a cellulose-based antibacterial biomimetic hydrogel containing TA, PVA, and AgNPs. After determining its antibacterial activity by bacteriostatic zone method, it was found that the hydrogel was an effective antibacterial agent. The bacteriostatic zone diameters of *Escherichia coli* (*E. coli*) and *S. aureus* were 7.2 mm and 6.8 mm, respectively, when the addition of AgNPs-TA-cellulose nanocrystals in the hydrogel was 4.0 wt%, showing good bacteriostatic effects. In another study, Bundjaja et al. [96] adopted cellulose carbamate hydrogel loaded with AgNPs and studied the effect of surfactant rarasaponin on the antibacterial activity of AgNPs cellulose carbamate hydrogel. It was found that the addition of surfactant helped to prevent the accumulation of AgNPs and increased the dispersion of AgNPs compared with the hydrogel without modified surfactant. It also increases the contact probability between AgNPs and bacteria, thus increasing the antibacterial activity of the hydrogel. The antibacterial activity of the hydrogel was determined by bacteriostatic zone method, and it was found that the hydrogel had excellent antibacterial activity against *E. coli* and *S. aureus.* The cytotoxicity of hydrogel was evaluated by skin fibroblast L929, and it was found that hydrogel had good biocompatibility. Combined with its excellent antibacterial properties, the hydrogel was confirmed to be a promising wound dressing. In the meantime, some researchers adopted silver nanoclusters (AgNCs) with smaller particle size to provide antibacterial activity for composite hydrogel. Liu et al. [97] prepared an antibacterial cellulose hydrogel containing AgNCs by in-situ synthesis of AgNCs on the nanofibers of bacterial cellulose hydrogel. The antibacterial activity of AgNCs hydrogel with different loading doses was tested by bacteriostatic zone method using Gram-positive bacteria *S. aureus* as model bacteria. The results showed that the antibacterial effect of AgNCs hydrogel prepared by 10 mmol/L AgNO_3_ solution was the best. The amount of Ag loading of hydrogel under this condition was determined to be 11.25 mg/g by inductively coupled plasma mass spectrometry, and the diameter of bacteriostatic zone of hydrogel to *S. aureus* was 4 cm. In the meantime, it was also found that the antibacterial activity of the hydrogel prepared by this method against Gram-positive bacteria and Gram-negative bacteria was better than that of AgNCs alone.

Besides, different from simply using AgNPs, some researchers adopted aminated AgNPs (AgNPs-NH_2_) to provide antibacterial activity for composite hydrogel. For instance, Liu et al. [108] crosslinked aminated AgNPs, gelatin and carboxylated cellulose nanofibers through dynamic ion bridges to form hydrogel. The antibacterial activity of the hydrogel against *S. aureus* and *Pseudomonas aeruginosa* (*P. aeruginosa*) was analyzed by turbidimetry and bacteriostatic zone method. It was found that the hydrogel had inhibitory effect on the two most common bacteria, and the hydrogel containing 0.5 mg/mL AgNPs-NH_2_ had the best antibacterial effect. In addition, the hydrogel has the advantages of water retention, hemostasis, and good biocompatibility, which can promote wound healing.

In addition to using AgNPs to provide antibacterial activity for composite hydrogel, CuNPs were also used to provide antibacterial activity for composite hydrogel. Al-Enizi et al. [78] prepared cellulose hydrogel containing CuNPs by polymer cross-linking method. Bacteriostatic zone method was adopted to determine the antibacterial activity of the hydrogel against pathogens of urinary tract infection. It was found that the antibacterial activity of hydrogel increased with the increase of CuNPs loading. When the concentration of CuNPs in the hydrogel is 5 mg/mL, the diameter of the bacteriostatic zone to *E. coli*, *Klebsiella pneumoniae* (*K. pneumoniae*), *P. aeruginosa*, *Proteus vulgaris*, *S. aureus* and *Proteus mirabilis* is 16.0 mm, 15.2 mm, 16.4 mm, 15.8 mm, 15.6 mm and 15.8 mm, respectively. The experimental results showed that the composite hydrogel had excellent bacteriostatic effect. In the meantime, it was found that the hydrogel also had good biocompatibility by evaluating the cytotoxicity of the hydrogel to human cervical cancer cell line. As a consequence, based on the water absorption, antibacterial, and biocompatibility of the hydrogel, the hydrogel has the potential to be developed into sanitary napkins, diapers, and other related care products.

In general, some metal nanoparticles can bring high efficiency, low drug resistance and broad-spectrum antibacterial activity to cellulose-based hydrogels. Cellulose-based hydrogels containing metal nanoparticles, as an antibacterial material, indeed have ideal antibacterial effect and has a wide range of applications. However, the high cost of preparation, long-term biological toxicity, and environmental cumulative toxicity of metal nanoparticles are still problems that need to be further clarified and solved. Simultaneously, the stability and durability of the antibacterial effect of hydrogels are associated with the even distribution and long-term stable presence of nanometals in hydrogels, which need to be further discovered. Additionally, most metal nanoparticles are presently bonded to cellulose-based hydrogels by physical interaction, which is not powerful enough and cannot be fixed-point modified. Consequently, developing more reliable and controllable modification methods in the subsequent research is required.

### 3.2. Cellulose-Based Antibacterial Hydrogels Loaded with Metal Oxide Nanoparticles

**Figure 3:**
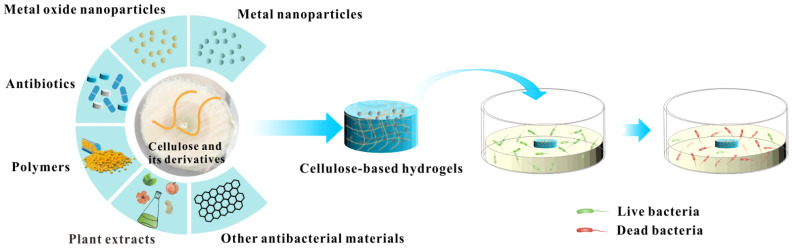
Antibacterial schematic diagram of cellulose-based hydrogels loaded with different antibacterial agents.

In addition to the antibacterial properties brought by the combination of metal nanoparticles and cellulose-based hydrogels, as the further development of nanoscience and nanotechnology, it has been reported that metal oxide nanoparticles, antibiotics, polymers plant extracts and other antibacterial materials are combined with cellulose-based hydrogels (Figure 3). Metal oxides (such as ZnO, TiO_2_, and CuO) act as enhancers for cellulose-based hydrogels and provide excellent antibacterial properties for cellulose composite hydrogels. The inclusion of metal oxides increased the density of the hydrogel network. In the meantime, the dense hydrogel network turns into a nanoscale reservoir layer for drugs, further improving the effect of hydrogel loading and slow-release antibacterial agents, then extending the antibacterial timeliness of hydrogels [109]. Similar to metal nanoparticles, metal oxides also have broad-spectrum antibacterial and low drug resistance. Moreover, metal oxide nanoparticles have stronger chemical stability and antioxidant capacity [110,111,112,113,114,115].

Zinc oxide nanoparticles (ZnONPs) were developed to provide antibacterial activity for cellulose-based hydrogels. For example, Yadollahi et al. [116] prepared carboxymethyl cellulose-ZnONPs composite hydrogel by in-situ synthesis of ZnONPs in expanded carboxymethyl cellulose hydrogel. The antibacterial activity of the hydrogel against *E. coli* and *S. aureus* was tested by bacteriostatic zone method. The antibacterial circle diameter of the hydrogel to *E. coli* (20 ± 2 mm) and *S. aureus* (28 ± 2 mm) is the largest when the zinc nitrate concentration is 0.03 mol/L. It has been proven that the hydrogel has excellent antibacterial activity against these two kinds of bacteria, and its antibacterial activity increases with the increase of ZnONPs concentration in the hydrogel. 

In another study, George et al. [117] prepare cellulose composite hydrogel containing ZnONPs using crosslinked dialdehyde cellulose prepared from sugarcane cellulose with chitosan, and mixed with ZnONPs synthesized from muskmelon seed extract. The antibacterial potential of the hydrogel was determined by bacteriostatic zone method, and its antibacterial activity against common skin infection pathogens *S. aureus* and *Trichophyton rubrum* was studied. It was found that the chitosan cellulose hydrogel without ZnONPs had certain antibacterial properties, and the antibacterial activity came from chitosan. In contrast, it was found that the antibacterial activity of the hydrogel added with ZnONPs was significantly improved. Besides, it was also found that the hydrogel can be adopted as a carrier of curcumin, and the loading rate can reach at 89.68%. In the meantime, the antibacterial activity of curcumin is well preserved.

Tungsten oxide nanoparticles (WO_3_NPs) were adopted to provide antibacterial activity for cellulose-based hydrogels. Based on hydroxyethyl cellulose and adding WO_3_NPs, Fawal et al. [40] prepared a new WO_3_NPs-hydroxyethyl cellulose hydrogel by casting method for wound treatment. The wound healing activity of the hydrogel was studied by human dermal fibroblast scratch test. The biocompatibility, anti-inflammatory activity, and antibacterial activity of the hydrogel were tested by methyl thiazolyl tetrazolium (MTT) method, enzyme-linked immunosorbent assay and bacteriostatic circle method. The results showed that the hydrogel had good wound healing activity, biocompatibility, anti-inflammatory activity and antibacterial effect. The diameter of the bacteriostatic zone against the five kinds of *Shigella* sp., *Salmonella* sp., *P. aeruginosa*, *Bacillus cereus*, *S. aureus* was 19 ± 0.12 mm, 26 ± 0.2 mm, 20 ± 0.13 mm, 17 ± 0.15 mm, and 14 ± 0.13 mm, respectively, when the concentration of WO_3_NPs in the hydrogel was 0.08 wt%. Besides, it was found that the hydrogel increased the activity of human normal cells (leukocytes and human dermal fibroblasts) and reduced the cytotoxicity of WO_3_NPs. Combining these research results, it can be found that the hydrogel has the potential to become an excellent wound dressing.

Titanium dioxide nanoparticles (TiO_2_NPs) were used to provide antibacterial activity for cellulose-based hydrogels. TiO_2_NPs sol was synthesized by Zhang et al. [118] in β-cyclodextrin cellulose solution via sol-gel method, and then crosslinked the mixture with epichlorohydrin to prepare a new type of TiO_2_NPs-β-cyclodextrin-cellulose hydrogel composites. Using *E. coli* and *S. aureus* as model bacteria, the antibacterial activity of the hydrogel was investigated under natural light and dark conditions, respectively. Curcumin was adopted as a model drug to test the sustained release behavior of curcumin in phosphate buffer. The results showed that the hydrogel had good antibacterial activity under light condition, but its antibacterial activity was negligible under no light condition. In the meantime, through the drug sustained release experiment, it was found that curcumin could be completely released from the hydrogel after 120 h.

Copper oxide nanoparticles (CuONPs) were exploited to provide antibacterial activity for composite hydrogel. Dharmalingam et al. [119] combined sodium carboxymethyl cellulose and hydroxypropyl methyl cellulose with CuONPs to form cellulose composite hydrogel using citric acid as non-toxic cross-linking agent. The bacteriostatic activity of the hydrogel against *S. aureus* and *E. coli* was determined by the bacteriostatic zone method. It was found that, the bacteriostatic effect was the best, and the diameter of bacteriostatic zone for *S. aureus* and *E. coli* was about 12 mm when the addition of citric acid in the hydrogel was 20%. In the meantime, it was also found that the hydrogel had biocompatibility for the proliferation of HaCaT cells. 

Similar to metal nanoparticles, cellulose-based hydrogels containing metal oxide nanoparticles has high efficiency, low drug resistance and broad-spectrum antibacterial properties. As a consequence, it has a very broad application prospect. However, for metal oxide nanoparticles, the problems of long-term biological toxicity and environmental cumulative toxicity are also that need to be further clarified and solved. Despite the fact that metal oxide nanoparticles are more challenging to be oxidized and more stable than metal nanoparticles, metal oxide nanoparticles have the disadvantage of being harder to release metal ions. This drawback will diminish the antibacterial effect of metal oxide nanoparticles, so how to make metal oxide nanoparticles that can release metal ions in hydrogels consistently and steadily deserves further investigation. Correspondingly, metal oxide nanoparticles also have the disadvantage of easy aggregation, and new modification methods need to be developed to overcome them. Thus, one of the advantages of metal oxide nanoparticles in antibacterial aspects also needs to be thoroughly exploited. That is, metal oxide nanoparticles are more prone to producing reactive oxygen species (ROS) than metal nanoparticles. This is to say that ROS have an awesome antibacterial effect, so it is very worthy of attention and further study. 

### 3.3. Cellulose-Based Antibacterial Hydrogels Loaded with Antibiotics

Since the 20th century, antibiotics have been the first choice for the treatment of bacterial infections due to their efficient bactericidal ability and low toxicity to mammalian cells [120]. The application of antibiotics is one of the most exciting events in modern medicine, which has saved countless lives and greatly extended the life span of human beings [121]. Direct use of antibiotics may be an effective way to combat many infections, but direct use of antibiotics will have adverse factors such as environmental toxicity, bacterial drug resistance, short duration of antibacterial activity, out of control of local concentration, and degradation in some application scenarios [120,122,123,124,125]. Thus, it is necessary to design a drug delivery system with high biocompatibility and good antibacterial effect, which not only meets the requirements of low cytotoxicity, but also meets the antibacterial requirements [122,126,127].

Linezolid was adopted to provide antibacterial activity for cellulose-based hydrogels. Drug-loaded composite hydrogel was prepared by Forero-Doria et al. [128] from cross-linking cellulose, chalcone and carbon nanotubes. With a single antibiotic as positive control, the inhibitory activity of linezolid-loaded hydrogel on *Enterococcus faecalis* (*E. faecium*) was studied. The results showed that the antibacterial effect of antibiotics was better in the first hour during the bacteriostatic process. However, with the passage of time, it would lose its effectiveness, and the antibacterial time of the composite hydrogel loaded with linezolid on *E. faecium* could be up to 48 h. It was found that the hydrogel can enhance the stability of linezolid after the combination of linezolid and hydrogel, and slowly release it in the solution to play a stronger antibacterial effect. The above findings showed that the drug-loaded composite hydrogel prepared by this method had great potential in promoting wound healing.

Tetracycline was adopted to provide antibacterial activity for cellulose-based hydrogels. Nanocomposite hydrogel containing polyethylene glycol, acrylamide, N, N’-methylene bisacrylamide and cellulose nanofibers was prepared by Iman et al. [129], and then loaded tetracycline into the hydrogel. The bacteriostatic effect of the hydrogel on *S. aureus* and *E. coli* was determined by bacteriostatic zone method. It was found that the pure hydrogel without tetracycline had no antibacterial activity. When the content of tetracycline was 300 μg/g, the antibacterial circle diameter of the hydrogel against *S. aureus* and *E. coli* was 33 mm and 25 mm, respectively. The hydrogel containing 4 wt% cellulose nanofibers have the best controlled release effect on tetracycline and does not destroy the stability of tetracycline. Besides, it was found that the hydrogel had good biocompatibility by testing the toxicity of hydrogel to the intestines and stomach of rats.

Minocycline hydrochloride was adopted to provide antibacterial activity for cellulose-based hydrogels. Patwa et al. [130] prepared a magnetic cellulose composite hydrogel using ion cross-linking between alginate and casein and doping bacterial cellulose modified by magnetic nanoparticles. Minocycline hydrochloride was loaded into the composite hydrogel as a model drug, and the composite hydrogel modified by minocycline hydrochloride was prepared. The bacteriostatic effect of the hydrogel on *E. coli* and *S. aureus* was investigated by bacteriostatic zone test. It was found that the hydrogel had obvious inhibitory effect on *S. aureus* and *E. coli* at 24 h, 48 h, one week, and two weeks. Through the drug-controlled release experiment, it was found that the hydrogel could release antibiotics continuously and effectively for a long time (more than two weeks). The cytotoxicity of hydrogel was evaluated by mouse embryonic fibroblasts and it was found that the hydrogel did not have any cytotoxicity and was suitable for transdermal administration.

Clindamycin was developed to provide antibacterial activity for cellulose-based hydrogels. Sadeghi et al. [131] prepared cellulose composite hydrogel with sustained release effect on clindamycin using carboxymethyl cellulose and human hair keratin as raw materials, citric acid as cross-linking agent with halloysite nanotubes and clindamycin. The antibacterial activity of the hydrogel against *S. aureus* was evaluated by bacteriostatic zone method and colony counting method. The obtained results showed that the antibacterial activity of the hydrogel was the highest when the ratio of keratin to carboxymethyl cellulose in the hydrogel was 0:1, the antibacterial rate against *S. aureus* was 99.66 ± 0.7%, and had antibacterial persistence (more than 24 h). Besides, it was also found that hydrogel could significantly promote the attachment, proliferation and diffusion of fibroblasts with the increase of keratin content. It can be found that the composite hydrogel is a promising candidate material to promote skin tissue repair and regeneration.

Herbmedotcin was adopted to provide antibacterial activity for cellulose-based hydrogels. Johnson et al. [132] adopted cellulose nanofibers and κ-carrageenan oligosaccharide nanoparticles as raw materials to prepare Herbmedotcin-κ-carrageenan oligosaccharide-cellulose composite hydrogel, which were adopted to kill periodontitis-associated bacteria. The antibacterial activity of the hydrogel against *Streptococcus mutans*, *Porphyromonas gingivalis*, *Fusobacterium nucleatum*, *and P. aeruginosa* was tested by the bacteriostatic zone method. It was found that the hydrogel had strong antibacterial activity against the above four kinds of bacteria, and the bacteriostatic effect was the best when the loading amount of Herbmedotcin in the hydrogel was 4 mg/mL. The diameter of the corresponding bacteriostatic zone was *Streptococcus mutans* 26.33 ± 1.52 mm, *Porphyromonas gingivalis* 18.33 ± 0.57 mm, *Fusobacterium nucleatum* 20.33 ± 0.63 mm, *and P. aeruginosa* 20.66 ± 1.25 mm. Additionally, hydrogel also reduced the production of ROS in gingival fibroblasts of patients with periodontitis, indicating that hydrogel has antibacterial and anti-inflammatory properties and has the potential to be adopted in the treatment of periodontitis.

Up to now, antibiotics are still the first choice to fight bacterial infection in the biomedical field, protecting human health. However, antibiotics also have fatal shortcomings, resulting in the emergence of a large number of drug-resistant bacteria, which is a serious threat to human health and health safety (Table 3). In the follow-up study on the combination of antibiotics and cellulose hydrogel, on the one hand, it is necessary to pay attention to the development of antibiotics with better antibacterial effect and lower drug resistance. On the other hand, it is necessary to systematically study the effects of the combination of antibiotics and cellulose hydrogel on the stability of antibiotics, antibacterial properties, and bacterial resistance, as well as potential environmental toxicity.

### 3.4. Cellulose-Based Antibacterial Hydrogels Loaded with Polymers

In the past few decades, polymer nanomaterials have controllable structure and properties, and have been widely adopted in biomedical and other fields with the development of polymer chemistry [153]. Natural polymers and synthetic polymers generally have high availability, good biocompatibility and biodegradability. As a consequence, they are a kind of new materials that have attracted much attention [154,155,156]. Polymers containing antibacterial functional groups are widely adopted to improve the efficacy of existing antibacterial agents, reduce the impact of traditional antibacterial agents on the environment and prolong the service life of antibacterial agents. Moreover, since antibacterial polymers are the next generation of biological fungicides, it is helpful to delay bacterial drug resistance [157]. Besides, the combination of antibacterial polymers with cellulose hydrogel can achieve effective loading and local sustained release of polymers and improve their solubility, biodistribution, and stability in vitro and in vivo [123,158].

Polyhexamethylene guanidine hydrochloride was adopted to provide antibacterial activity for cellulose-based hydrogels. Pan et al. [159] modified bagasse cellulose with polyhexamethylene guanidine hydrochloride, and then crosslinked the modified cellulose with unmodified cellulose in different proportions to synthesize a new type of green antibacterial cellulose composite hydrogel dressing. The inhibitory effect of hydrogel on *E. coli* was studied by colony count method and bacteriostatic zone method. The growth inhibition rate of the hydrogel grafted with 1.5 wt% polyhexamethylene guanidine hydrochloride on *E. coli* was 99%. In the meantime, the covalent bonding of antibacterial polymer and cellulose main chain eliminates the harmful effect on the environment due to the no leaching effect, which also ensures the durability of the antibacterial effect of hydrogel. Besides, the retention rate of cell activity of hydrogel to NIH3T3 cells was more than 76%, which proved that the biocompatibility of hydrogel was good. As a consequence, the new green antibacterial hydrogel prepared by this method is considered to be an ideal candidate for chronic wound dressing and skin tissue repair.

Polyamidoxime was adopted to provide antibacterial activity for cellulose-based hydrogels. Gao et al. [160] prepared a cellulose polyamidoxime composite hydrogel by UV polymerization. The quaternary ammonium group of allyltrimethylamine in hydrogel has broad-spectrum antibacterial activity. Through bacteriostatic experiment, it was found that the inhibition rate of hydrogel on *S. aureus*, *E. coli and Vibrio alginolyticus* was 74.2 ± 5.27%, 84.1 ± 4.31% and 69.2 ± 5.76%, respectively. Furthermore, the hydrogel not only has excellent antibacterial properties, but also has high water/ion conductivity and strong uranium adsorption capacity.

The ε-poly-L-lysine (PLL)was adopted to provide antibacterial activity for cellulose-based hydrogels. Tavakolian et al. [80] prepared a carboxylated cellulose hydrogel carrying PLL by covalent cross-linking of carboxylated cellulose with natural antibacterial agent PLL. The antibacterial properties of the hydrogel against *P. aeruginosa* and *S. aureus* were tested by a variety of bacteriostatic experiments. The results showed that the hydrogel containing 200 mg/L PLL could successfully inhibit the growth of *P. aeruginosa* and *S. aureus*, and the minimum inhibitory concentrations (MIC) of *P. aeruginosa* was lower than that of *S. aureus*, since the peptidoglycan layer of Gram-positive bacteria *P. aeruginosa* was thicker and harder. It was necessary to contact with antibacterial agents more fully to destroy the cell wall of bacteria. In the meantime, it was also found that the hydrogel killed 99.5% and 98.5% of *P. aeruginosa* and *S. aureus* within three hours, respectively, and could fully inhibit the formation of bacterial membrane. The cellulose-containing hydrogel containing PLL prepared by this method has good antibacterial and water absorption, so it is a promising candidate material for wound dressing.

Using schizophyllan to provide antibacterial activity for cellulose-based hydrogels, Hamedi et al. [161] prepared a new cellulose composite hydrogel composed of amino bacterial cellulose and natural polymers of schizophyllan. The antibacterial activity of the hydrogel was studied by colony counting method. It was found that the inhibition rates of *E. coli* and *S. aureus* of the composite hydrogel containing 1 wt% schizophyllan were 50% and 30%, respectively. In the meantime, the combination of bacterial cellulose and schizophyllan polymer provides excellent thermal stability, water retention and mechanical strength for the hydrogel. Besides, the study also found that the hydrogel can stimulate the proliferation of human fibroblasts.

Polyethyleneimine (PEI) was adopted to provide antibacterial activity for cellulose-based hydrogels. Wahid et al. [162] developed a polyethyleneimine-bacterial cellulose antibacterial hydrogel using epichlorohydrin as coupling agent. The antibacterial properties of hydrogel against *S. aureus* and *E. coli* were determined by bacteriostatic zone method and colony counting method. The results showed that the antibacterial activity of hydrogel was related to the content of PEI. The antibacterial activity of hydrogel against two kinds of bacteria was more than 99% when the amount of PEI in hydrogel was ≥12.88 mg/mL. Besides, hydrogel also shows good thermal properties, compressibility and plasticity. As a result, it has a broad application prospect in the field of biomedicine.

Dehydrogenative polymer of coniferyl alcohol was adopted to provide antibacterial activity for composite hydrogel. Zmejkoski et al. [163] designed a composite hydrogel composed of bacterial cellulose and dehydrogenative polymer of coniferyl alcohol for the treatment of chronic wounds. By studying the antibacterial activity of hydrogel against *P.aeruginosa*, *Salmonella typhimurium*, *Listeria monocytogenes* and *S. aureus*, it was found that hydrogel had inhibitory effect on the above four kinds of bacteria. In addition, the study also found that hydrogel has high detumescence and good slow-release effect on dehydrogenative polymer of coniferyl alcohol, which clearly proves that hydrogel is a promising chronic wound healing agent.

Povidone iodine was adopted to provide antibacterial activity for cellulose-based hydrogels. Jantrawut et al. [164] combined low methoxy pectin, gelatin and carboxymethyl cellulose to prepare cellulose composite hydrogel, and 10% povidone iodine was added to the hydrogel. The antibacterial activity of hydrogel against *S. aureus* was tested by bacteriostatic zone test. The results showed that the diameter of bacteriostatic zone of hydrogel loaded with 10% povidone iodine against *S. aureus* was 22.06 ± 3.44 mm, which was stronger than that of hydrogel and iodine solution. In the meantime, the study also found that the hydrogel has high water absorption, water retention and air permeability, indicating that the hydrogel is expected to become an efficient wound dressing.

Antibacterial polymers generally have good biocompatibility and degradability. Synthetic polymers and natural polymers are diverse, which is an important treasure house for the development of new and excellent antibacterial agents. In the meantime, polymers generally have strong modifiability and can be covalently linked with cellulose-based hydrogels, which enhance the stability and dispersibility of polymers on the one hand, and improve the properties of cellulose-based hydrogels on the other hand. As a consequence, constantly looking for new polymer antibacterial agents and combining with cellulose-based hydrogels is a direction worthy of in-depth study.

### 3.5. Cellulose-Based Antibacterial Hydrogels Loaded with Plant Extracts

For a long time, plant extracts have been the key research object in the search for new antibacterial agents, and many botanical antibacterial agents have been developed [165]. In addition, the proportion of application of plant extracts in clinical treatment is getting higher and higher [166]. Especially in the face of the severe threat of antibiotic-resistant bacteria, plant extracts show great potential to treat drug-resistant bacteria, and they are safe, effective, and environmentally friendly. Plant extracts have become a valuable source for the development of new and efficient antibacterial compounds [167,168].

Gallic acid was adopted to provide antibacterial activity for cellulose-based hydrogels. Pinho et al. [169] crosslinked β-cyclodextrin with hydroxypropyl methyl cellulose under mild conditions and doped gallic acid to prepare gallic acid-β-cyclodextrin-cellulose composite hydrogel. By testing the antibacterial activity of hydrogel against *Staphylococcus epidermidis*, *S. aureus*, and *K. pneumoniae*, it was found that gallic acid concentration in hydrogel could significantly inhibit the growth of these three bacteria when the concentration of gallic acid in hydrogel reached 0.26 mmol/L. 

**Figure 4:**
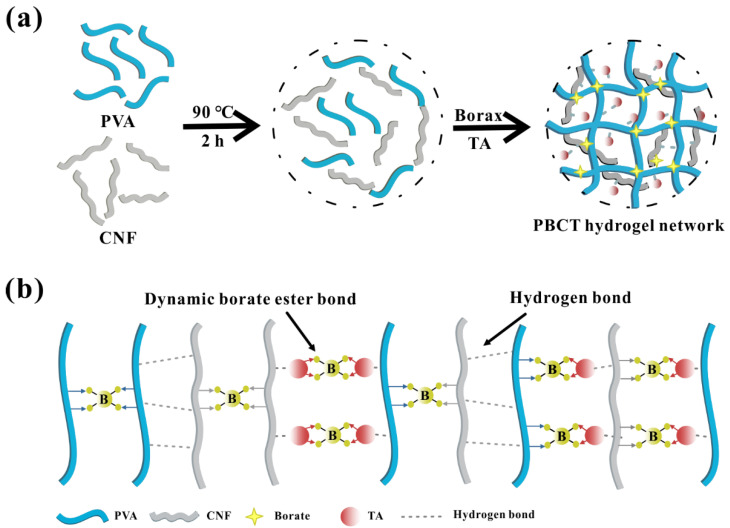
Scheme for synthesis of tannic acid-polyvinyl alcohol-cellulose composite hydrogel (**a**) and the chemical bond diagram in the hydrogel (**b**) (reprinted from reference [170] with permission from Elsevier).

TA was adopted to provide antibacterial activity for cellulose-based hydrogels. Ge et al. [170] crosslinked cellulose nanofibers with PVA using borax and mixed TA with natural product TA to obtain TA-PVA-cellulose composite hydrogel (Figure 4a). Inside the hydrogel, cellulose nanofibers and polyvinyl alcohol are crosslinked by dynamic borate eater bond and hydrogen bond (Figure 4b). Bacteriostatic zone method was adopted to determine the antibacterial activity of hydrogel against *S. aureus*. It was found that the diameter of inhibition zone of hydrogel against *S. aureus*. increased with the increase of TA content. The bacteriostatic effect of TA:PVA = 20:1 *w*/*w* in the hydrogel is the best, and the diameter of the antibacterial circle of the hydrogel to *S. aureus*. is about 7 mm. Besides, it was also found that the hydrogel has the advantages of good oxidation resistance, high extensibility, plasticity and rapid self-healing.

Using isoliquiritigenin to provide antibacterial activity for cellulose-based hydrogels, hyaluronic acid-hydroxyethyl cellulose composite hydrogel doped with isoliquiritigenin prepared by chemical cross-linking method as reported by Kwon et al. [171]. It was found that the hydrogel had the best rheological and adhesion properties when the mass ratio of hyaluronic acid to hydroxyethyl cellulose was 3:1. In the meantime, the hydrogel had the best 70% release rate of isoliquiritigenin when pH = 7 was used. Through colony counting method, it was found that the hydrogel had good inhibitory activity on *Propionibacterium acnes*. Besides, the hydrogel also showed good skin hair follicle permeability of isoliquiritigenin. These results show that the pH-sensitive hydrogel is an effective transdermal antibacterial agent and has the potential to be adopted in the treatment of acne.

Grapefruit seed extract was adopted to provide antibacterial activity for cellulose-based hydrogels. Koneru et al. [49] crosslinked sodium carboxymethyl cellulose and hydroxypropyl methyl cellulose with citric acid and mixed with grapefruit seed extract to prepare cellulose composite hydrogel containing grapefruit seed extract. The antibacterial activity of the hydrogel against *E. coli* and *S. aureus* was tested by bacteriostatic zone method. It was found that the hydrogel had ideal antibacterial rate against two kinds of bacteria, and the antibacterial activity against *E. coli* was higher than that against *S. aureus*. The antibacterial circle diameter against *E. coli* and *S. aureus* was about 12 mm and 11 mm, respectively, when the content of grapefruit extract in the hydrogel was 0.015 wt%.

Thymol was adopted to provide antibacterial activity for cellulose-based hydrogels. The bacterial cellulose composite hydrogel rich in thymol was prepared as reported by Jiji et al. [172]. The inhibitory effect of the hydrogel on *E. coli and S. aureus*, *P. aeruginosa*, and *K. pneumoniae* was analyzed by bacteriostatic zone method. It was found that the bacterial cellulose hydrogel treated in 10 mL 1% thymol solution showed significant inhibitory activity against three kinds of bacteria, and the diameter of bacteriostatic zone was *E. coli* 18.33 ± 1.15 mm, *S. aureus* 40.33 ± 1.15 mm, *P. aeruginosa* 20.67 ± 0.57 mm, and *K. pneumoniae* 41.33 ± 1.15 mm. In particular, the inhibitory effect on *K. pneumoniae* and *S. aureus* is better than that of gentamicin. In addition, the results of in vivo studies show that hydrogel can accelerate wound healing and is an ideal wound dressing.

With the increasingly serious threat of drug-resistant bacteria, plant-derived antibacterial components show lower drug resistance in the antibacterial process, which brings hope for dealing with the harm of drug-resistant bacteria. Moreover, plant extracts have the advantages of rich resources, wide variety, low toxicity and convenient extraction, and have the potential to provide a large number of new antibacterial agents with high efficiency, broad spectrum and low drug resistance for human beings. Looking for new antibacterial agents from plant extracts is a significant and valuable research direction. The combination of plant extract and cellulose-based hydrogels will be beneficial to the dissolution, structural stability and controlled release of plant extracts, which is of great significance to broaden the application range of plant extracts antibacterial agents and improve its antibacterial effect.

### 3.6. Cellulose-Based Antibacterial Hydrogels Loaded with Other Materials

Apart from the antibacterial properties of cellulose-based hydrogels loaded with metal and their oxide nanoparticles, antibiotics, polymers and plant extracts, there are also some studies on the addition of other antibacterial materials to cellulose-based hydrogels. For example, using biopolyesters to provide antibacterial activity for cellulose-based hydrogels, Virginia Rivero-Buceta et al. [173] prepared antibacterial biological polyesters bacterial cellulose composite hydrogel using bacterial fibers and antibacterial biological polyesters (3-hydroxyacetylthioalkanoic acid-co-3-hydroxyalkanoic acid, PHACOS) as raw materials, and tested the antibacterial activity of the hydrogel against *S. aureus*. It was found that when the mass fraction of PHACOS in the hydrogel is 20%, the hydrogel can kill more than 95% *S. aureus*, and the hydrogel has good mechanical and thermal properties, and can effectively promote wound healing. Besides, fibroblasts were adopted to test the cytotoxicity of hydrogel, and it was found that the hydrogel could maintain more than 85% of the cell activity after seven days.

Cationic dendrimer was adopted to provide antibacterial activity for cellulose-based hydrogels. Fan et al. [174] prepared a cationic dendrimer-carboxylated cellulose nanofibers hydrogel. By testing the MIC and minimum bactericidal concentrations (MBC) of the hydrogel, it was found that when the connection order of cationic dendrimer was three or four, the killing rate of *E. coli*, *S. aureus* and *P. aeruginosa* was close to 100%. In addition, its germicidal efficacy comes from the slow release of dendrimers in the hydrogel. Besides, the study also discovered that the hydrogel has good biocompatibility.

**Figure 5:**
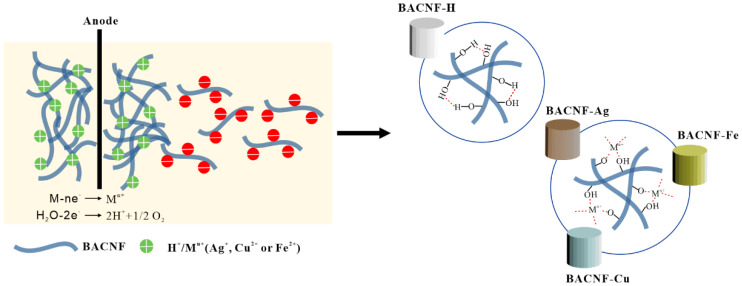
The scheme for synthesis carboxyl cellulose composite hydrogels containing silver, copper and iron ions was prepared by electrochemical method (reprinted from reference [176] with permission from Elsevier).

Some metal salts or metal ions were adopted to provide antibacterial activity for cellulose-based hydrogels. Du et al. [175] prepared silver sulfadiazine-chitosan-cellulose composite hydrogel by the reaction of carboxymethyl chitosan with Schiff base of oxidized carboxymethyl cellulose and doping with silver sulfadiazine (AgSD). The antibacterial properties of the hydrogel against *S. aureus* and *E. coli* were well studied by bacteriostatic zone method. It was found that the hydrogel had inhibitory effect on both bacteria, and there was a slight bacteriostatic zone (*E. coli* 1.91 ± 0.10 mm, *S. aureus* 1.69 ± 0. 19 mm) around the hydrogel when the content of AgSD was 2 mg/mL, which may be due to the non-adhesion of bacteria caused by negative charge. Moreover, the negatively charged hydrogel has low adhesion and can effectively reduce the secondary trauma when changing the wound dressing when adopted as a wound dressing. In another study, Guo et al. [176] prepared brown algae carboxyl cellulose nanofiber (BACNF) composite hydrogel containing silver, copper and iron, respectively, using electrochemical methods to involve metal ions in the cross-linking of cellulose (Figure 5). The bacteriostatic effect of silver, copper and iron ion cellulose composite hydrogel was tested by bacteriostatic zone method, and it was found that the cellulose composite hydrogel containing silver and copper had better inhibitory effect on *E. coli* and *S. aureus*. In addition, it was also found that the composite hydrogel prepared by this method had interconnected nanopore structure, which enhanced the thermal degradation ability, mechanical strength and antibacterial activity of the hydrogel.

Reduced graphene oxide was adopted to provide antibacterial activity for cellulose-based hydrogels. Ali et al. [177] prepared the reduced graphene oxide-sodium carboxymethyl cellulose composite hydrogel by adding reduced graphene oxide into the hydrogel. Through testing the antibacterial activity of hydrogel against *S. aureus* and *P. aeruginosa*, it was found that the hydrogel containing reduced graphene oxide 100 ug/mL could successfully inhibit the formation of *S. aureus* and *P. aeruginosa* biofilm, indicating that the hydrogel has the potential to be adopted in the treatment of biofilm-related infections.

Quaternary ammonium salts were adopted to provide antibacterial activity for cellulose-based hydrogels. Wang et al. [178] prepared mesoporous silica foam-quaternized hydroxyethyl cellulose composite hydrogel with hemostatic and antibacterial effects by doping mesoporous silica foam and free radical graft copolymerization. The antibacterial activity of the hydrogel against *E. coli* and *S. aureus* was evaluated by colony counting method. The results showed that the hydrogel had excellent antibacterial properties against *E. coli* and *S. aureus*, and the bactericidal rate was more than 99%. Besides, when the concentration of quaternary ammonium group in the hydrogel was 2.732 mmol/g, the hydrogel not only had significant antibacterial activity, but also had good cytocompatibility and efficient hemostatic effect, indicating that the hydrogel has the potential to be adopted in wound healing.

It can combine with a variety of nanoparticles, polymers, macromolecules, small molecules, or ions to form composite hydrogel since cellulose-based hydrogels have high modifiability, including chemical modification and physical doping. As a consequence, on the basis of existing research, it is still necessary to continue to look for new antibacterial agents to combine with cellulose-based hydrogels. The purpose of this study is to improve the properties of cellulose-based hydrogels, broaden the application range of antibacterial agents, or improve the antibacterial effect of antibacterial agents.

## 4. Antibacterial Mechanism of Cellulose-Based Composite Hydrogels

The antibacterial mechanism is determined by its own antibacterial agents since cellulose-based hydrogels itself do not have antibacterial activity [57]. An antibacterial agent often has multiple antibacterial mechanisms, and the multiple mechanisms together lead to bacterial death. Moreover, the antibacterial mechanisms of different types of antibacterial agents will be somewhat diverse [57]. In summary, it is found that the antibacterial mechanism of cellulose-based antibacterial hydrogel can be roughly divided into three types. First, antibacterial properties are achieved by destroying the cell membrane of bacteria [179]. Secondly, antibacterial properties are achieved by affecting bacterial protein activity or DNA synthesis [131]. Third, antibacterial properties are achieved by producing ROS that cause oxidative damage to bacteria [119].

To most antibacterial agents, the antibacterial mechanism is disrupting the cell membrane of bacteria [117,180], which is also the most prevalent antibacterial mechanism. Al-Enizi et al. [78] prepared cellulose hydrogel containing CuNPs, it was found that the antibacterial activity of hydrogel increased with the increase of CuNPs loading. CuNPs and Cu^2+^ released from the composite hydrogel can react with the hydrophobic membrane of bacterial cells, affecting a variety of biological activities of the cell membrane, thereby damaging the cell. Su et al. [179] adopted carboxymethyl cellulose composite hydrogel containing tetracycline hydrochloride to resist bacterial. It was found that after bacteria reacted with hydrogel, the shape and size of bacteria changed, the surface of bacteria was wrinkled and damaged, and there was leakage of cell contents. Wahid et al. [181] used a bacterial cellulose complex hydrogel containing chitosan to resist bacterial, the interaction between positively charged chitosan and negatively charged bacterial cell membrane, which increases the permeability of cell membrane and inhibits bacterial growth. Koneru et al. [49] prepared cellulose composite hydrogel containing grapefruit seed extract. They used composite hydrogel mixed with grapefruit seed extract for antibacterial. It was found that the antibacterial activity of the hydrogel came from the polyphenols in the grapefruit extract. These polyphenols caused bacterial death by destroying the peptide bonds in the bacterial cell membrane.

Antibacterial is achieved by affecting bacterial protein activity or DNA synthesis. The principle is that the activity of the protein will be reduced or lost when the antibacterial agents bind to protein or DNA, and the replication of DNA will not be able to proceed [182,183]. Khamrai et al. [184] found that curcumin inhibits bacterial proliferation via interfering with the synthesis of proteins necessary for bacterial division by using bacterial cellulose composite hydrogels loaded with curcumin. Sadeghi et al. [131] used a cellulose composite hydrogel loaded with clindamycin for antibacterial, and found that clindamycin in the hydrogel inhibited the synthesis of bacterial protein and destroyed the bacterial cell membrane by combining with the large subunit of bacterial ribosome, resulting in bacterial death. Similarly, Wahid et al. [181] found that there are two antibacterial mechanisms of chitosan in the antibacterial process by using bacterial cellulose composite hydrogel containing chitosan. In addition to inhibiting bacterial growth by disrupting the bacterial cell membrane. The other one is that positively charged chitosan binds to bacterial DNA to inhibit the production of mRNA in bacteria, resulting in bacterial death. In addition, when AgNPs and CuNPs act as antibacterial agents, there are also two ways to hinder DNA synthesis and inactivate proteins by binding with some cellular proteins in their antibacterial mechanism, and the realization of these two ways is mainly completed by Ag^+^ and Cu^2+^ released by AgNPs and CuNPs, respectively [78,182].

**Figure 6:**
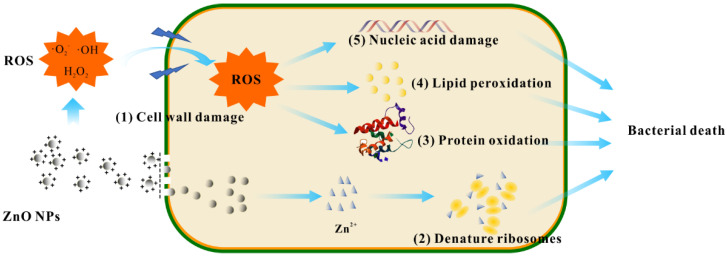
The antibacterial mechanism diagram of cellulose composite hydrogel loaded with ZnONPs.

Antibacterial is achieved by producing ROS that cause oxidative damage to bacteria. ROS oxidize certain proteins, lipids, or nucleic acids in bacteria, resulting in bacterial death [185]. This antibacterial mechanism is rare in the antibacterial process of cellulose-based hydrogels, and is mainly concentrated in the research reports using metal nanoparticles or metal oxide nanoparticles as antibacterial agents [119,186]. Yadollahi et al. [116] adopted carboxymethyl cellulose composite hydrogel containing ZnONPs to resist bacteria. It was found that the antibacterial property of the hydrogel may be related to the photocatalytic mechanism of ZnONPs. ZnONPs produces hydrogen peroxide under photocatalysis, which leads to the destruction of the structure and function of bacterial cell membrane (Figure 6). Dharmalingam et al. [119] used CuONPs-containing hydroxypropyl methyl cellulose composite hydrogel to be antibacterial, and found that the antibacterial mechanism of the hydrogels may be due to the ROS generated by CuONPs that make the lipids in the bacterial cell membrane peroxidation, resulting in bacterial death. Sarkandi et al. [101] prepared a AgNPs-bacterial cellulose composite hydrogel and studied the antibacterial activity of the hydrogel. It was found that AgNPs formed ROS by releasing Ag^+^, resulting in oxidative damage to bacteria and thus death.

Through the analysis of the above research reports, it can be found that no matter which antibacterial agents are adopted to provide antibacterial activity for cellulose-based hydrogels, the most common antibacterial mechanism is that antibacterial agents destroy the cell membrane of bacteria and thus cause the permeability of the cell membrane to change or directly break, leading to the death of bacteria. The least antibacterial mechanism is to produce ROS to cause oxidative damage for bacteria. Further analysis of the antibacterial mechanism of different antibacterial agents demonstrated that metal nanoparticles possess the three antibacterial mechanisms mentioned above at the same time, which is rarely possessed among other antibacterial agents (and which is also the reason for the best antibacterial effect and lowest bacterial resistance). In future research, the antibacterial mechanism of cellulose-based hydrogels should be further studied, and the similarities and differences between antibacterial mechanisms before and after combining with cellulose-based hydrogels should be explored. In the meantime, it is also necessary to refer to the antibacterial mechanism of cellulose-based hydrogels to evaluate whether cellulose-based hydrogels will cause similar damage to normal human cells.

## 5. Conclusions and Prospect

Many important advances have been made in the preparation of various cellulose-based nanomaterials from cellulose. In particular, cellulose has been proved to be a high-quality raw material for the preparation of hydrogel due to its unique nanostructure, excellent mechanical properties, biodegradability, and biocompatibility. In addition, cellulose-based hydrogels have also been proved to be excellent biomaterials for carrying and slow-releasing a variety of antibacterial agents. The applications and characteristics of several typical cellulose-based antibacterial hydrogels, including cellulose-based hydrogels loaded with metal nanoparticles, metal oxide nanoparticles, antibiotics, polymers, and plant extracts, were summarized, and the latest progress in the antibacterial mechanisms of cellulose-based hydrogels were reviewed in detail. In addition, the preparation methods of cellulose-based composite hydrogels were also introduced. This is expected to provide valuable typical cases and theoretical references for the research and application of functionalized cellulose-based antibacterial hydrogels.

**Figure 7:**
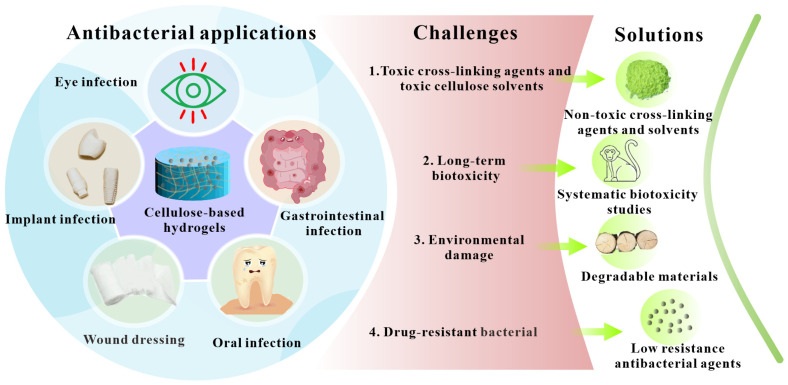
Challenges and solutions in antibacterial applications of cellulose-based hydrogels.

Through the previous discussion, it can be found that cellulose-based antibacterial hydrogels have broad application prospects, especially in the biomedical field. However, there are still many challenges to be overcome before its large-scale commercial application (Figure 7): (1) green and non-toxic cellulose solvents and cross-linking agents should be developed for the synthesis of cellulose-based hydrogels; (2) more efficient and environmentally friendly methods should be developed toward synthesizing cellulose-based hydrogels with particular applications; (3) before the clinical application of cellulose-based antibacterial hydrogels, its long-term biological safety should be systematically evaluated in small animal models (such as mice, rats) and large animal models (such as monkeys); (4) the integrated use of antibiotics and cellulose-based hydrogels should be defined and it should be determined whether this will lead to a more severe crisis of drug-resistant bacteria and figure and what the corresponding countermeasures should be; (5) for cellulose-based antibacterial hydrogels containing inorganic nanoparticles, it is necessary to fully investigate the potential toxicity and final clearance mechanism of those nanoparticles in human body; and (6) in addition to paying attention to the application of cellulose-based antibacterial hydrogels in biomedical and related fields, attention should also be paid to its applications and potential risks in the agriculture, environment, food and other industries.

## Figures and Tables

**Table 1:** Advantages and disadvantages of different cellulose derivatives for the preparation of cellulose-based hydrogels.

Cellulose Derivatives	Advantages	Disadvantages	Typical Applications	References
Methyl cellulose	Water retention, good stability and biosafety	Easy to precipitate in salt solution	Biocomposite film Food packaging	[26,27,28,29,30]
Carboxymethyl cellulose	Excellent tackifying effect, biocompatible and biodegradable	Sensitive to the pH, temperature and ionic strength of solution	Hydrogel beads Metal ions removal	[31,32,33,34,35]
Hydroxyethyl cellulose	Biocompatible, excellent tackifying effect and highly water retention	Prone to degradation and instability	Wound dressing Self-healing hydrogel	[36,37,38,39,40,41]
Hydroxypropyl cellulose	Stability, non-biotoxicity and water-soluble	Potential hypersensitivity	Thermoresponsive hydrogel Biomedical	[23,24,25,42,43]
Hydroxypropyl methyl cellulose	Excellent film-forming and dispersion, water retention	Potential skin irritation	Scaffolds materials Drug delivery	[44,45,46,47,48,49]

**Table 2:** Representative examples of cellulose-based hydrogels loaded with AgNPs.

Cellulose-Based Hydrogels	Bacterial	Applications	References
AgNPs-carboxymethyl cellulose hydrogel	*S. aureus* *E. coli*	Biomaterial	[98]
AgNPs-gelatin-cellulose hydrogel	*E. coli*	Wound healing in nursing care of infants	[99]
AgNPs-alginate-nanocrystalline cellulose hydrogel	*S. aureus* *P. aeruginosa*	Active materials for clinical applications	[100]
AgNPs-cellulose carbamate hydrogel	*E. coli*	Wound dressing	[96]
	*S. aureus*		
AgNPs-bacterial cellulose nanocomposite hydrogel	*S. aureus* *E. coli*	Wound dressing	[101]
AgNPs-polyvinyl alcohol-bacterial cellulose hydrogel	*E. coli* *S. aureus*	Wound treatment	[102]
AgNPs-polyacrylamide-hydroxyethyl cellulose hydrogel	*E. coli* *S. aureus*	Antibacterial strain sensor	[103]
AgNPs-carboxymethyl cellulose hydrogel	*E. coli* *S. aureus*	Antibacterial material	[104]
AgNPs-phthalated cashew gum-carboxymethyl cellulose hydrogel	*S. aureus* *P. aeruginosa*	Wound treatment	[105]
AgNPs-polyacrylic acid-cellulose hydrogel	*E. coli*	Antibacterial material	[106]
AgNPs-carboxymethyl cellulose hydrogel	*S. aureus* *P. aeruginosa*	Antibacterial material	[107]

**Table 3:** The advantages and disadvantages of different antibacterial agents used to modify cellulose-based hydrogels.

Antibacterial Agents	Advantages	Disadvantages	Typical Applications	References
Metal or oxidized metal nanoparticles	Broad-spectrum and long-term antibacterial, low bacterial resistance	Tend to agglomerate, certain cytotoxicity and environmental toxicity	Wound dressing Self-healing artificial skin	[91,95,133,134,135,136,137]
Antibiotics	Specific and efficient antibacterial, ideal biocompatibility	Bacterial resistance and short-term antibacterial activity, prone to degradation and instability	Clinical antibacterial Wound healing	[120,122,138,139,140,141]
Polymers	Biodegradable and nontoxic, high modifiability and biocompatibility	Poor permeability	Antibacterial food packaging Treatment of periodontitis	[132,142,143,144,145,146]
Plant extracts	Rich resources, environmentally friendly, anti-drug resistant bacteria	Difficult to extract and enrich	Biomedicine Treatment of chronic infection	[147,148,149,150,151,152]

## Data Availability

Not applicable.

## References

[1] Su H.-C., Liu Y.-S., Pan C.-G., Chen J., He L.-Y., Ying G.-G. (2018). Persistence of antibiotic resistance genes and bacterial community changes in drinking water treatment system: From drinking water source to tap water. Sci. Total Environ..

[2] Hultman J., Rahkila R., Ali J., Rousu J., Bjorkroth K.J. (2015). Meat processing plant microbiome and contamination patterns of cold-tolerant bacteria causing food safety and spoilage risks in the manufacture of vacuum-packaged cooked sausages. Appl. Environ. Microbiol..

[3] Mugoyela V., Mwambete K.D. (2010). Microbial contamination of nonsterile pharmaceuticals in public hospital settings. Ther. Clin. Risk Manag..

[4] Jernigan J.A., Hatfield K.M., Wolford H., Nelson R.E., Olubajo B., Reddy S.C., McCarthy N., Paul P., McDonald L.C., Kallen A. (2020). Multidrug-resistant bacterial infections in US hospitalized patients, 2012–2017. N. Engl. J. Med..

[5] Aminov R.I. (2010). A brief history of the antibiotic era: Lessons learned and challenges for the future. Front. Microbiol..

[6] Willing B.P., Russell S.L., Finlay B.B. (2011). Shifting the balance: Antibiotic effects on host-microbiota mutualism. Nat. Rev. Microbiol..

[7] Li S., Dong S., Xu W., Tu S., Yan L., Zhao C., Ding J., Chen X. (2018). Antibacterial hydrogels. Adv. Sci..

[8] Davies J., Davies D. (2010). Origins and evolution of antibiotic resistance. Microbiol. Mol. Biol. Rev..

[9] Piddock L.J.V. (2012). The crisis of no new antibiotics-what is the way forward?. Lancet Infect. Dis..

[10] Ahmed E.M. (2015). Hydrogel: Preparation, characterization, and applications: A review. J. Adv. Res..

[11] Qian H., Wang J., Yan L. (2020). Synthesis of lignin-poly(N-methylaniline)-reduced graphene oxide hydrogel for organic dye and lead ions removal. J. Bioresour. Bioprod..

[12] Zhang A., Wang F., Chen L., Wei X., Xue M., Yang F., Jiang S. (2021). 3D printing hydrogels for actuators: A review. Chin. Chem. Lett..

[13] Li P., Poon Y.F., Li W.F., Zhu H.Y., Yeap S.H., Cao Y., Qi X.B., Zhou C.C., Lamrani M., Beuerman R.W. (2011). A polycationic antimicrobial and biocompatible hydrogel with microbe membrane suctioning ability. Nat. Mater..

[14] Guan G., Zhang S.-Y., Cai Y., Liu S., Bharathi M.S., Low M., Yu Y., Xie J., Zheng Y., Zhang Y.-W. (2014). Convenient purification of gold clusters by co-precipitation for improved sensing of hydrogen peroxide, mercury ions and pesticides. Chem. Commun..

[15] Khalil H.P.S.A., Davoudpour Y., Islam M.N., Mustapha A., Sudesh K., Dungani R., Jawaid M. (2014). Production and modification of nanofibrillated cellulose using various mechanical processes: A review. Carbohydr. Polym..

[16] Navarra M.A., Dal Bosco C., Moreno J.S., Vitucci F.M., Paolone A., Panero S. (2015). Synthesis and characterization of cellulose-based hydrogels to be used as gel electrolytes. Membranes.

[17] Moon R.J., Martini A., Nairn J., Simonsen J., Youngblood J. (2011). Cellulose nanomaterials review: Structure, properties and nanocomposites. Chem. Soc. Rev..

[18] Altomare L., Cochis A., Carletta A., Rimondini L., Fare S. (2016). Thermo-responsive methylcellulose hydrogels as temporary substrate for cell sheet biofabrication. J. Mater. Sci. -Mater. Med..

[19] Pandey J.K., Takagi H., Nakagaito A.N., Saini D.R., Ahn S.-H. (2012). An overview on the cellulose based conducting composites. Compos. Part B-Eng..

[20] Pandey J.K., Ahn S.H., Lee C.S., Mohanty A.K., Misra M. (2010). Recent advances in the application of natural fiber based composites. Macromol. Mater. Eng..

[21] Joseph B., Sagarika V.K., Sabu C., Kalarikkal N., Thomas S. (2020). Cellulose nanocomposites: Fabrication and biomedical applications. J. Bioresour. Bioprod..

[22] Prusty K., Swain S.K. (2021). Polypropylene oxide/polyethylene oxide-cellulose hybrid nanocomposite hydrogels as drug delivery vehicle. J. Appl. Polym. Sci..

[23] Morishima R., Bokuda K. (2021). Hydroxypropyl cellulose-induced fixed drug eruption as an adverse effect of generic drugs. Contact Dermat..

[24] Lisuzzo L., Caruso M.R., Cavallaro G., Milioto S., Lazzara G. (2021). Hydroxypropyl cellulose films filled with halloysite nanotubes/wax hybrid microspheres. Ind. Eng. Chem. Res..

[25] De Moura M.R., Mattoso L.H.C., Zucolotto V. (2012). Development of cellulose-based bactericidal nanocomposites containing silver nanoparticles and their use as active food packaging. J. Food Eng..

[26] Pan X., Zhuang H., Zhang L., Wang J., Wang S. (2018). Preparation and characterIzation of methyl cellulose/poly (gamma-glutamic acid) composite hydrogel. Ion Exch. Adsorpt..

[27] Bampidis V., Azimonti G., Bastos M.L., Christensen H., Dusemund B., Durjava M.K., Kouba M., Lopez-Alonso M., Puente S.L., Marcon F. (2020). Safety and efficacy of methyl cellulose for all animal species. EFSA J..

[28] Wilhelmy C., Pavez C., Bordeu E., Brossard N. (2021). A review of tannin determination methods using spectrophotometric detection in red wines and their ability to predict astringency. S. Afr. J. Enol. Vitic..

[29] Lagaron J.M., Fendler A. (2009). High water barrier nanobiocomposites of methyl cellulose and chitosan for film and coating applications. J. Plast. Film. Sheeting.

[30] de Dicastillo C.L., Bustos F., Guarda A., Jose Galotto M. (2016). Cross-linked methyl cellulose films with murta fruit extract for antioxidant and antimicrobial active food packaging. Food Hydrocoll..

[31] Liu K., Du H., Zheng T., Liu H., Zhang M., Zhang R., Li H., Xie H., Zhang X., Ma M. (2021). Recent advances in cellulose and its derivatives for oilfield applications. Carbohydr. Polym..

[32] Dai H.J., Huang H.H. (2017). Enhanced swelling and responsive properties of pineapple peel carboxymethyl cellulose-g-poly(acrylic acid-co-acrylamide) superabsorbent hydrogel by the introduction of carclazyte. J. Agric. Food Chem..

[33] Liu C., Omer A.M., Ouyang X.K. (2018). Adsorptive removal of cationic methylene blue dye using carboxymethyl cellulose/k-carrageenan/activated montmorillonite composite beads: Isotherm and kinetic studies. Int. J. Biol. Macromol..

[34] Benhalima T., Ferfera-Harrar H., Lerari D. (2017). Optimization of carboxymethyl cellulose hydrogels beads generated by an anionic surfactant micelle templating for cationic dye uptake: Swelling, sorption and reusability studies. Int. J. Biol. Macromol..

[35] Tran Thu H., Okabe H., Hidaka Y., Hara K. (2017). Removal of metal ions from aqueous solutions using carboxymethyl cellulose/sodium styrene sulfonate gels prepared by radiation grafting. Carbohydr. Polym..

[36] Seddiqi H., Oliaei E., Honarkar H., Jin J.F., Geonzon L.C., Bacabac R.G., Klein-Nulend J. (2021). Cellulose and its derivatives: Towards biomedical applications. Cellulose.

[37] Umoren S.A., Eduok U.M. (2016). Application of carbohydrate polymers as corrosion inhibitors for metal substrates in different media: A review. Carbohydr. Polym..

[38] Morales J.O., McConville J.T. (2011). Manufacture and characterization of mucoadhesive buccal films. Eur. J. Pharm. Biopharm..

[39] Diao Y., Song M., Zhang Y., Shi L.-y., Lv Y., Ran R. (2017). Enzymic degradation of hydroxyethyl cellulose and analysis of the substitution pattern along the polysaccharide chain. Carbohydr. Polym..

[40] El Fawal G.F., Abu-Serie M.M., Hassan M.A., Elnouby M.S. (2018). Hydroxyethyl cellulose hydrogel for wound dressing: Fabrication, characterization and in vitro evaluation. Int. J. Biol. Macromol..

[41] Hussain I., Sayed S.M., Liu S., Yao F., Oderinde O., Fu G. (2018). Hydroxyethyl cellulose-based self-healing hydrogels with enhanced mechanical properties via metal-ligand bond interactions. Eur. Polym. J..

[42] Dai L., Cheng T., Duan C., Zhao W., Zhang W.P., Zou X.J., Aspler J., Ni Y.H. (2019). 3D printing using plant-derived cellulose and its derivatives: A review. Carbohydr. Polym..

[43] Yuan M., Bi B., Huang J., Zhuo R., Jiang X. (2018). Thermosensitive and photocrosslinkable hydroxypropyl chitin-based hydrogels for biomedical applications. Carbohydr. Polym..

[44] Popov T.A., Aberg N., Emberlin J., Josling P., Ilyina N.I., Nikitin N.P., Church M. (2017). Methyl-cellulose powder for prevention and management of nasal symptoms. Expert Rev. Respir. Med..

[45] Bampidis V., Azimonti G., Bastos M.d.L., Christensen H., Dusemund B., Durjava M.K., Kouba M., Lopez-Alonso M., Puente S.L., Marcon F. (2020). Safety and efficacy of hydroxypropyl methyl cellulose for all animal species. EFSA J..

[46] Gupta B., Mishra V., Gharat S., Momin M., Omri A. (2021). Cellulosic polymers for enhancing drug bioavailability in ocular drug delivery systems. Pharmaceuticals.

[47] Obara S., Muto H., Kokubo H., Ichikawa N., Kawanabe M., Tanaka O. (1992). Primary dermal and eye irritability tests of hydrophobically modified hydroxypropyl methylcellulose in rabbits. J. Toxicol. Sci..

[48] Zeeshan R., Mutahir Z., Iqbal H., Ali M., Iqbal F., Ijaz K., Sharif F., Shah A.T., Chaudhry A.A., Yar M. (2018). Hydroxypropylmethyl cellulose (HPMC) crosslinked chitosan (CH) based scaffolds containing bioactive glass (BG) and zinc oxide (ZnO) for alveolar bone repair. Carbohydr. Polym..

[49] Koneru A., Dharmalingam K., Anandalakshmi R. (2020). Cellulose based nanocomposite hydrogel films consisting of sodium carboxymethylcellulose-grapefruit seed extract nanoparticles for potential wound healing applications. Int. J. Biol. Macromol..

[50] Vinatier C., Gauthier O., Fatimi A., Merceron C., Masson M., Moreau A., Moreau F., Fellah B., Weiss P., Guicheux J. (2009). An injectable cellulose-based hydrogel for the transfer of autologous nasal chondrocytes in articular cartilage defects. Biotechnol. Bioeng..

[51] Chang C., Duan B., Cai J., Zhang L. (2010). Superabsorbent hydrogels based on cellulose for smart swelling and controllable delivery. Eur. Polym. J..

[52] Frazier T., Alarcon A., Wu X., Mohiuddin O.A., Motherwell J.M., Carlsson A.H., Christy R.J., Edwards J.V., Mackin R.T., Prevost N. (2020). Clinical translational potential in skin wound regeneration for sdipose-ferived, blood-derived, and cellulose materials: Cells, exosomes, and hydrogels. Biomolecules.

[53] Tong R., Chen G., Pan D., Qi H., Li R.A., Tian J., Lu F., He M. (2019). Highly stretchable and compressible cellulose ionic hydrogels for flexible strain sensors. Biomacromolecules.

[54] Bauli C.R., Lima G.F., de Souza A.G., Ferreira R.R., Rosa D.S. (2021). Eco-friendly carboxymethyl cellulose hydrogels filled with nanocellulose or nanoclays for agriculture applications as soil conditioning and nutrient carrier and their impact on cucumber growing. Colloids Surf. A-Physicochem. Eng. Asp..

[55] Zhou D., Zhang L., Guo S.L. (2005). Mechanisms of lead biosorption on cellulose/chitin beads. Water Res..

[56] Xiong X.P., Zhang L., Wang Y.F. (2005). Polymer fractionation using chromatographic column packed with novel regenerated cellulose beads modified with silane. J. Chromatogr. A.

[57] Alavi M. (2019). Modifications of microcrystalline cellulose (MCC), nanofibrillated cellulose (NFC), and nanocrystalline cellulose (NCC) for antimicrobial and wound healing applications. E-Polymers.

[58] Wang F., Pan Y., Cai P., Guo T., Xiao H. (2017). Single and binary adsorption of heavy metal ions from aqueous solutions using sugarcane cellulose-based adsorbent. Bioresour. Technol..

[59] Sabbagh F., Muhamad I.I., Pa’e N., Hashim Z. (2018). Strategies in improving properties of cellulose-based hydrogels for smart applications. Cellulose-Based Superabsorbent Hydrogels.

[60] Kabir S.M.F., Sikdar P.P., Haque B., Bhuiyan M.A.R., Ali A., Islam M.N. (2018). Cellulose-based hydrogel materials: Chemistry, properties and their prospective applications. Prog. Biomater..

[61] Mohamad N., Loh E.Y.X., Fauzi M.B., Ng M.H., Amin M.C.I.M. (2019). In vivo evaluation of bacterial cellulose/acrylic acid wound dressing hydrogel containing keratinocytes and fibroblasts for burn wounds. Drug Deliv. Transl. Res..

[62] Zhang Y., Xu A., Lu B., Li Z., Wang J. (2015). Dissolution of cellulose in 1-allyl-3-methylimizodalium carboxylates at room temperature: A structure property relationship study. Carbohydr. Polym..

[63] Gonzalez-Torres M., Leyva-Gomez G., Rivera M., Krotzsch E., Rodriguez-Talavera R., Leonor Rivera A., Cabrera-Wrooman A. (2018). Biological activity of radiation-induced collagen-polyvinylpyrrolidone-PEG hydrogels. Mater. Lett..

[64] Zainal S.H., Mohd N.H., Suhaili N., Anuar F.H., Lazim A.M., Othaman R. (2021). Preparation of cellulose-based hydrogel: A review. J. Mater. Res. Technol.-JmrT.

[65] Zhao D., Huang J., Zhong Y., Li K., Zhang L., Cai J. (2016). High-strength and high-toughness double-cross-linked cellulose hydrogels: A new strategy using sequential chemical and physical cross-linking. Adv. Funct. Mater..

[66] Shen X., Shamshina J.L., Berton P., Gurau G., Rogers R.D. (2016). Hydrogels based on cellulose and chitin: Fabrication, properties, and applications. Green Chem..

[67] Chang C., Zhang L. (2011). Cellulose-based hydrogels: Present status and application prospects. Carbohydr. Polym..

[68] Wang L.-Y., Wang M.-J. (2016). Removal of heavy metal ions by poly (vinyl alcohol) and carboxymethyl cellulose composite hydrogels prepared by a freeze-thaw method. ACS Sustain. Chem. Eng..

[69] Yang J., Han C.-R., Duan J.-F., Xu F., Sun R.-C. (2013). Mechanical and viscoelastic properties of cellulose nanocrystals reinforced poly (ethylene glycol) nanocomposite hydrogels. ACS Appl. Mater. Interfaces.

[70] Ibrahim S.M., El Salmawi K.M., Zahran A.H. (2007). Synthesis of crosslinked superabsorbent carboxymethyl cellulose/acrylamide hydrogels through electron-beam irradiation. J. Appl. Polym. Sci..

[71] Kim Y., Kim Y.K., Kim S., Harbottle D., Lee J.W. (2017). Nanostructured potassium copper hexacyanoferrate-cellulose hydrogel for selective and rapid cesium adsorption. Chem. Eng. J..

[72] Ooi S.Y., Ahmad I., Amin M.C.I.M. (2016). Cellulose nanocrystals extracted from rice husks as a reinforcing material in gelatin hydrogels for use in controlled drug delivery systems. Ind. Crops Prod..

[73] Wang Z., Fan X., He M., Chen Z., Wang Y., Ye Q., Zhang H., Zhang L. (2014). Construction of cellulose-phosphor hybrid hydrogels and their application for bioimaging. J. Mater. Chem. B.

[74] Fu L.-H., Qi C., Ma M.-G., Wan P. (2019). Multifunctional cellulose-based hydrogels for biomedical applications. J. Mater. Chem. B.

[75] Demitri C., Del Sole R., Scalera F., Sannino A., Vasapollo G., Maffezzoli A., Ambrosio L., Nicolais L. (2008). Novel superabsorbent cellulose-based hydrogels crosslinked with citric acid. J. Appl. Polym. Sci..

[76] Yan L., Shuai Q., Gong X., Gu Q., Yu H. (2009). Synthesis of microporous cationic hydrogel of hydroxypropyl cellulose (HPC) and its application on anionic dye removal. Clean-Soil Air Water.

[77] Marsano E., Bianchi E., Sciutto L. (2003). Microporous thermally sensitive hydrogels based on hydroxypropyl cellulose crosslinked with poly-ethyleneglicol diglycidyl ether. Polymer.

[78] Al-Enizi A.M., Ahamad T., Al-Hajji A.B., Ahmed J., Chaudhary A.A., Alshehri S.M. (2018). Cellulose gum and copper nanoparticles based hydrogel as antimicrobial agents against urinary tract infection (UTI) pathogens. Int. J. Biol. Macromol..

[79] He M., Zhao Y., Duan J., Wang Z., Chen Y., Zhang L. (2014). Fast contact of solid-liquid interface created high strength multi-layered cellulose hydrogels with controllable size. ACS Appl. Mater. Interfaces.

[80] Tavakolian M., Munguia-Lopez J.G., Valiei A., Islam M.S., Kinsella J.M., Tufenkji N., van de Ven T.G.M. (2020). Highly absorbent antibacterial and biofilm-disrupting hydrogels from cellulose for wound dressing applications. ACS Appl. Mater. Interfaces.

[81] Oyarzun-Ampuero F., Vidal A., Concha M., Morales J., Orellana S., Moreno-Villoslada I. (2015). Nanoparticles for the treatment of wounds. Curr. Pharm. Des..

[82] Liang D., Lu Z., Yang H., Gao J., Chen R. (2016). Novel asymmetric wettable AgNPs/chitosan wound dressing: In vitro and in vivo evaluation. ACS Appl. Mater. Interfaces.

[83] Mokhena T.C., Luyt A.S. (2017). Electrospun alginate nanofibres impregnated with silver nanoparticles: Preparation, morphology and antibacterial properties. Carbohydr. Polym..

[84] Shao W., Wu J., Wang S., Huang M., Liu X., Zhang R. (2017). Construction of silver sulfadiazine loaded chitosan composite sponges as potential wound dressings. Carbohydr. Polym..

[85] Bao Y., He J., Song K., Guo J., Zhou X., Liu S. (2021). Plant-extract-mediated synthesis of metal nanoparticles. J. Chem..

[86] Celebioglu A., Aytac Z., Umu O.C.O., Dana A., Tekinay T., Uyar T. (2014). One-step synthesis of size-tunable Ag nanoparticles incorporated in electrospun PVA/cyclodextrin nanofibers. Carbohydr. Polym..

[87] Csoka L., Bozanic D.K., Nagy V., Dimitrijevic-Brankovic S., Luyt A.S., Grozdits G., Djokovic V. (2012). Viscoelastic properties and antimicrobial activity of cellulose fiber sheets impregnated with Ag nanoparticles. Carbohydr. Polym..

[88] Emam H.E., Mowafi S., Mashaly H.M., Rehan M. (2014). Production of antibacterial colored viscose fibers using in situ prepared spherical Ag nanoparticles. Carbohydr. Polym..

[89] Ilic V., Saponjic Z., Vodnik V., Potkonjak B., Jovancic P., Nedeljkovic J., Radetic M. (2009). The influence of silver content on antimicrobial activity and color of cotton fabrics functionalized with Ag nanoparticles. Carbohydr. Polym..

[90] Lavorgna M., Attianese I., Buonocore G.G., Conte A., Del Nobile M.A., Tescione F., Amendola E. (2014). MMT-supported Ag nanoparticles for chitosan nanocomposites: Structural properties and antibacterial activity. Carbohydr. Polym..

[91] Gondikas A.P., Morris A., Reinsch B.C., Marinakos S.M., Lowry G.V., Hsu-Kim H. (2012). Cysteine-induced modifications of zero-valent silver nanomaterials: Implications for particle surface chemistry, aggregation, dissolution, and silver speciation. Environ. Sci. Technol..

[92] Qu F., Ding Y., Lv X., Xia L., You J., Han W. (2019). Emissions of terbium metal-organic frameworks modulated by dispersive/agglomerated gold nanoparticles for the construction of prostate-specific antigen biosensor. Anal. Bioanal. Chem..

[93] Jain A.S., Pawar P.S., Sarkar A., Junnuthula V., Dyawanapelly S. (2021). Bionanofactories for green synthesis of silver nanoparticles: Toward antimicrobial applications. Int. J. Mol. Sci..

[94] Tripathi D., Modi A., Narayan G., Rai S.P. (2019). Green and cost effective synthesis of silver nanoparticles from endangered medicinal plant Withania coagulans and their potential biomedical properties. Mater. Sci. Eng. C-Mater. Biol. Appl..

[95] Lin F., Wang Z., Shen Y., Tang L., Zhang P., Wang Y., Chen Y., Huang B., Lu B. (2019). Natural skin-inspired versatile cellulose biomimetic hydrogels. J. Mater. Chem. A.

[96] Bundjaja V., Santoso S.P., Angkawijaya A.E., Yuliana M., Soetaredjo F.E., Ismadji S., Ayucitra A., Gunarto C., Ju Y.-H., Ho M.-H. (2021). Fabrication of cellulose carbamate hydrogel-dressing with rarasaponin surfactant for enhancing adsorption of silver nanoparticles and antibacterial activity. Mater. Sci. Eng. C-Mater. Biol. Appl..

[97] Liu Y., Wang S., Wang Z., Yao Q., Fang S., Zhou X., Yuan X., Xie J. (2020). The in situ synthesis of silver nanoclusters inside a bacterial cellulose hydrogel for antibacterial applications. J. Mater. Chem. B.

[98] Park J.-S., Kuang J., Lim Y.-M., Gwon H.-J., Nho Y.-C. (2012). Characterization of silver nanoparticle in the carboxymethyl cellulose hydrogel prepared by a gamma ray irradiation. J. Nanosci. Nanotechnol..

[99] Gou L., Xiang M., Ni X. (2020). Development of wound therapy in nursing care of infants by using injectable gelatin-cellulose composite hydrogel incorporated with silver nanoparticles. Mater. Lett..

[100] Bergonzi C., Remaggi G., Graiff C., Bergamonti L., Potenza M., Ossiprandi M.C., Zanotti I., Bernini F., Bettini R., Elviri L. (2020). Three-dimensional (3D) printed silver nanoparticles/alginate/nanocrystalline cellulose hydrogels: Study of the antimicrobial and cytotoxicity efficacy. Nanomaterials.

[101] Sarkandi A.F., Montazer M., Harifi T., Rad M.M. (2021). Innovative preparation of bacterial cellulose/silver nanocomposite hydrogels: In situ green synthesis, characterization, and antibacterial properties. J. Appl. Polym. Sci..

[102] Song S., Liu Z., Abubaker M.A., Ding L., Zhang J., Yang S., Fan Z. (2021). Antibacterial polyvinyl alcohol/bacterial cellulose/nano-silver hydrogels that effectively promote wound healing. Mater. Sci. Eng. C-Mater. Biol. Appl..

[103] Wang X., Wang Z., Wang X., Shi L., Ran R. (2021). Preparation of silver nanoparticles by solid-state redox route from hydroxyethyl cellulose for antibacterial strain sensor hydrogel. Carbohydr. Polym..

[104] Yadollahi M., Namazi H., Aghazadeh M. (2015). Antibacterial carboxymethyl cellulose/Ag nanocomposite hydrogels cross-linked with layered double hydroxides. Int. J. Biol. Macromol..

[105] Lustosa A.K.M.F., de Jesus Oliveira A.C., Quelemes P.V., Placido A., da Silva F.V., Oliveira I.S., de Almeida M.P., Amorim A.D.G.N., Delerue-Matos C., de Oliveira R.D.C.M. (2017). In situ synthesis of silver nanoparticles in a hydrogel of carboxymethyl cellulose with phthalated-cashew gum as a promising antibacterial and healing agent. Int. J. Mol. Sci..

[106] Bajpai S.K., Bajpai M., Gautam D. (2013). In situ formation of silver nanoparticles in regenerated cellulose-polyacrylic acid (RC-PAAc) hydrogels for antibacterial application. J. Macromol. Sci. Part A-Pure Appl. Chem..

[107] Abou-Yousef H., Kamel S. (2015). High efficiency antimicrobial cellulose-based nanocomposite hydrogels. J. Appl. Polym. Sci..

[108] Liu R., Dai L., Si C., Zeng Z. (2018). Antibacterial and hemostatic hydrogel via nanocomposite from cellulose nanofibers. Carbohydr. Polym..

[109] Sabbagh F., Muhamad I.I. (2017). Acrylamide-based hydrogel drug delivery systems: Release of acyclovir from MgO nanocomposite hydrogel. J. Taiwan Inst. Chem. Eng..

[110] Dizaj S.M., Lotfipour F., Barzegar-Jalali M., Zarrintan M.H., Adibkia K. (2014). Antimicrobial activity of the metals and metal oxide nanoparticles. Mater. Sci. Eng. C-Mater. Biol. Appl..

[111] Gupta V., Singh S., Rawat K., Bohidar H.B., Solanki P.R. (2014). Cytotoxicity and antimicrobial activity of transition metal oxide nanoparticles. Natl. Conf. Nanotechnol. Renew. Energy (NCNRE).

[112] Hrenovic J., Milenkovic J., Daneu N., Kepcija R.M., Rajic N. (2012). Antimicrobial activity of metal oxide nanoparticles supported onto natural clinoptilolite. Chemosphere.

[113] Azam A., Ahmed A.S., Oves M., Khan M.S., Habib S.S., Memic A. (2012). Antimicrobial activity of metal oxide nanoparticles against Gram-positive and Gram-negative bacteria: A comparative study. Int. J. Nanomed..

[114] Bhanjana G., Kumar N., Thakur R., Dilbaghi N., Kumar S. (2011). Antimicrobial activity of metal & metal oxide nanoparticles interfaced with ligand complexes of 8-hydroxyquinoline and alpha-amino acids. Int. Conf. Adv. Condens. Nano Mater. (ICACNM).

[115] Applerot G., Lipovsky A., Dror R., Perkas N., Nitzan Y., Lubart R., Gedanken A. (2009). Enhanced antibacterial activity of nanocrystalline ZnO due to increased ROS-mediated cell injury. Adv. Funct. Mater..

[116] Yadollahi M., Gholamali I., Namazi H., Aghazadeh M. (2015). Synthesis and characterization of antibacterial carboxymethyl cellulose/ZnO nanocomposite hydrogels. Int. J. Biol. Macromol..

[117] George D., Maheswari P.U., Sheriffa Begum K.M.M., Arthanareeswaran G. (2019). Biomass-derived dialdehyde cellulose cross-linked chitosan-based nanocomposite hydrogel with phytosynthesized zinc oxide nanoparticles for enhanced curcumin delivery and bioactivity. J. Agric. Food Chem..

[118] Zhang H., Zhu J., Hu Y., Chen A., Zhou L., Gao H., Liu Y., Liu S. (2019). Study on photocatalytic antibacterial and sustained-release properties of cellulose/TiO_2_/beta-CD composite hydrogel. J. Nanomater..

[119] Dharmalingam K., Bordoloi D., Kunnumakkara A.B., Anandalakshmi R. (2020). Preparation and characterization of cellulose-based nanocomposite hydrogel films containing CuO/Cu_2_O/Cu with antibacterial activity. J. Appl. Polym. Sci..

[120] Gao W., Chen Y., Zhang Y., Zhang Q., Zhang L. (2018). Nanoparticle-based local antimicrobial drug delivery. Adv. Drug Deliv. Rev..

[121] Wang S., Gao Y., Jin Q., Ji J. (2020). Emerging antibacterial nanomedicine for enhanced antibiotic therapy. Biomater. Sci..

[122] Hoque J., Bhattacharjee B., Prakash R.G., Paramanandham K., Haldar J. (2018). Dual function injectable hydrogel for controlled release of antibiotic and local antibacterial therapy. Biomacromolecules.

[123] Xiong M.-H., Bao Y., Yang X.-Z., Zhu Y.-H., Wang J. (2014). Delivery of antibiotics with polymeric particles. Adv. Drug Deliv. Rev..

[124] Taccone F.S., Bond O., Cavicchi F.Z., Hites M. (2016). Individualized antibiotic strategies. Curr. Opin. Anesthesiol..

[125] Felton T.W., Hope W.W., Roberts J.A. (2014). How severe is antibiotic pharmacokinetic variability in critically ill patients and what can be done about it?. Diagn. Microbiol. Infect. Dis..

[126] Sattari S., Tehrani A.D., Adeli M. (2018). pH-responsive hybrid hydrogels as antibacterial and drug delivery systems. Polymers.

[127] Xu W., Dong S., Han Y., Li S., Liu Y. (2018). Hydrogels as antibacterial biomaterials. Curr. Pharm. Des..

[128] Forero-Doria O., Polo E., Marican A., Guzman L., Venegas B., Vijayakumar S., Wehinger S., Guerrero M., Gallego J., Duran-Lara E.F. (2020). Supramolecular hydrogels based on cellulose for sustained release of therapeutic substances with antimicrobial and wound healing properties. Carbohydr. Polym..

[129] Iman M., Barati A., Safari S. (2019). Characterization, in vitro antibacterial activity, and toxicity for rat of tetracycline in a nanocomposite hydrogel based on PEG and cellulose. Cellulose.

[130] Patwa R., Zandraa O., Capakova Z., Saha N., Saha P. (2020). Effect of iron-oxide nanoparticles impregnated bacterial cellulose on overall properties of alginate/casein hydrogels: Potential injectable biomaterial for wound healing applications. Polymers.

[131] Sadeghi S., Nourmohammadi J., Ghaee A., Soleimani N. (2020). Carboxymethyl cellulose-human hair keratin hydrogel with controlled clindamycin release as antibacterial wound dressing. Int. J. Biol. Macromol..

[132] Johnson A., Kong F., Miao S., Lin H.V., Thomas S., Huang Y.C., Kong Z.L. (2020). Therapeutic effects of antibiotics loaded cellulose nanofiber and kappa-carrageenan oligosaccharide composite hydrogels for periodontitis treatment. Sci. Rep..

[133] Raza S., Matula K., Karon S., Paczesny J. (2021). Resistance and adaptation of bacteria to non-antibiotic antibacterial agents: Physical stressors, nanoparticles, and bacteriophages. Antibiotics.

[134] Fatima F., Siddiqui S., Khan W.A. (2021). Nanoparticles as novel emerging therapeutic antibacterial agents in the antibiotics resistant era. Biol. Trace Elem. Res..

[135] Levard C., Hotze E.M., Lowry G.V., Brown G.E. (2012). Environmental transformations of silver nanoparticles: Impact on stability and toxicity. Environ. Sci. Technol..

[136] Kai J., Zhou X. (2020). Preparation, characterization, and cytotoxicity evaluation of zinc oxide-bacterial cellulose-chitosan hydrogels for antibacterial dressing. Macromol. Chem. Phys..

[137] Raho R., Nhu Y.N., Zhang N., Jiang W., Sannino A., Liu H., Pollini M., Paladini F. (2020). Photo-assisted green synthesis of silver doped silk fibroin/carboxymethyl cellulose nanocomposite hydrogels for biomedical applications. Mater. Sci. Eng. C-Mater. Biol. Appl..

[138] Ur Rehman M.S., Rashid N., Ashfaq M., Saif A., Ahmad N., Han J.-I. (2015). Global risk of pharmaceutical contamination from highly populated developing countries. Chemosphere.

[139] Cheesman M.J., Ilanko A., Blonk B., Cock I.E. (2017). Developing new sntimicrobial therapies: Are synergistic combinations of plant extracts/compounds with conventional antibiotics the solution?. Pharmacogn. Rev..

[140] Khan F., Pham D.T.N., Oloketuyi S.F., Kim Y.-M. (2020). Antibiotics application strategies to control biofilm formation in pathogenic bacteria. Curr. Pharm. Biotechnol..

[141] Bajpai S.K., Pathak V., Soni B. (2015). Minocycline-loaded cellulose nano whiskers/poly(sodium acrylate) composite hydrogel films as wound dressing. Int. J. Biol. Macromol..

[142] Shukla S.C., Singh A., Pandey A.K., Mishra A. (2012). Review on production and medical applications of epsilon-polylysine. Biochem. Eng. J..

[143] Alavi M., Nokhodchi A. (2020). An overview on antimicrobial and wound healing properties of ZnO nanobiofilms, hydrogels, and bionanocomposites based on cellulose, chitosan, and alginate polymers. Carbohydr. Polym..

[144] Rinaudo M. (2006). Chitin and chitosan: Properties and applications. Prog. Polym. Sci..

[145] Sangaj N.S., Malshe V.C. (2004). Permeability of polymers in protective organic coatings. Prog. Org. Coat..

[146] Wang C., Wang L., Zhang Q., Cheng L., Yue H., Xia X., Zhou H. (2021). Preparation and characterization of apoacynum venetum cellulose nanofibers reinforced chitosan-based composite hydrogels. Colloids Surf. B-Biointerfaces.

[147] Raskin I., Ribnicky D.M., Komarnytsky S., Ilic N., Poulev A., Borisjuk N., Brinker A., Moreno D.A., Ripoll C., Yakoby N. (2002). Plants and human health in the twenty-first century. Trends Biotechnol..

[148] Felix G., Soto-Robles C.A., Nava E., Lugo-Medina E. (2021). Principal metabolites in extracts of different plants responsible for antibacterial effects. Chem. Res. Toxicol..

[149] Chen L., Pang Y., Luo Y., Cheng X., Lv B., Li C. (2021). Separation and purification of plant terpenoids from biotransformation. Eng. Life Sci..

[150] Zhu H.-G., Tang H.-Q., Cheng Y.-Q., Li Z.-G., Tong L.-T. (2021). Electrostatic separation technology for obtaining plant protein concentrates: A review. Trends Food Sci. Technol..

[151] Mahomoodally M.F., Aumeeruddy M.Z., Rengasamy K.R.R., Roshan S., Hammad S., Pandohee J., Hu X., Zengin G. (2021). Ginger and its active compounds in cancer therapy: From folk uses to nano-therapeutic applications. Semin. Cancer Biol..

[152] Gupta A., Briffa S.M., Swingler S., Gibson H., Kannappan V., Adamus G., Kowalczuk M., Martin C., Radecka I. (2020). Synthesis of silver nanoparticles using curcumin-cyclodextrins loaded into bacterial cellulose-based hydrogels for wound dressing applications. Biomacromolecules.

[153] Sadrearhami Z., Thuy-Khanh N., Namivandi-Zangeneh R., Jung K., Wong E.H.H., Boyer C. (2018). Recent advances in nitric oxide delivery for antimicrobial applications using polymer-based systems. J. Mater. Chem. B.

[154] Lv X., Liu Y., Song S., Tong C., Shi X., Zhao Y., Zhang J., Hou M. (2019). Influence of chitosan oligosaccharide on the gelling and wound healing properties of injectable hydrogels based on carboxymethyl chitosan/alginate polyelectrolyte complexes. Carbohydr. Polym..

[155] Rasool A., Ata S., Islam A. (2019). Stimuli responsive biopolymer (chitosan) based blend hydrogels for wound healing application. Carbohydr. Polym..

[156] Sajjad W., Khan T., Ul-Islam M., Khan R., Hussain Z., Khalid A., Wahid F. (2019). Development of modified montmorillonite-bacterial cellulose nanocomposites as a novel substitute for burn skin and tissue regeneration. Carbohydr. Polym..

[157] Kenawy E.-R., Worley S.D., Broughton R. (2007). The chemistry and applications of antimicrobial polymers: A state-of-the-art review. Biomacromolecules.

[158] Martin C., Low W.L., Gupta A., Amin M.C.I.M., Radecka I., Britland S.T., Raj P., Kenward K. (2015). Strategies for antimicrobial drug delivery to biofilm. Curr. Pharm. Des..

[159] Pan Y., Zhao X., Li X., Cai P. (2019). Green-based antimicrobial hydrogels prepared from bagasse cellulose as 3D-scaffolds for wound dressing. Polymers.

[160] Gao J., Yuan Y., Yu Q., Yan B., Qian Y., Wen J., Ma C., Jiang S., Wang X., Wang N. (2020). Bio-inspired antibacterial cellulose paper-poly(amidoxime) composite hydrogel for highly efficient uranium(vi) capture from seawater. Chem. Commun. (Camb).

[161] Hamedi S., Shojaosadati S.A., Najafi V., Alizadeh V. (2020). A novel double-network antibacterial hydrogel based on aminated bacterial cellulose and schizophyllan. Carbohydr. Polym..

[162] Wahid F., Bai H., Wang F.-P., Xie Y.-Y., Zhang Y.-W., Chu L.-Q., Jia S.-R., Zhong C. (2019). Facile synthesis of bacterial cellulose and polyethyleneimine based hybrid hydrogels for antibacterial applications. Cellulose.

[163] Zmejkoski D., Spasojevic D., Orlovska I., Kozyrovska N., Sokovic M., Glamoclija J., Dmitrovic S., Matovic B., Tasic N., Maksimovic V. (2018). Bacterial cellulose-lignin composite hydrogel as a promising agent in chronic wound healing. Int. J. Biol. Macromol..

[164] Jantrawut P., Bunrueangtha J., Suerthong J., Kantrong N. (2019). Fabrication and characterization of low methoxyl pectin/gelatin/carboxymethyl cellulose absorbent hydrogel film for wound dressing applications. Materials.

[165] Upadhyay A., Mooyottu S., Yin H., Nair M.S., Bhattaram V., Venkitanarayanan K. (2015). Inhibiting microbial toxins using plant-derived compounds and plant extracts. Medicines.

[166] Antunes J.C., Domingues J.M., Miranda C.S., Silva A.F.G., Homem N.C., Amorim M.T.P., Felgueiras H.P. (2021). Bioactivity of chitosan-based particles loaded with plant-derived extracts for biomedical applications: Emphasis on antimicrobial fiber-based systems. Mar. Drugs.

[167] Friedman M. (2015). Antibiotic-resistant bacteria: Prevalence in food and inactivation by food-compatible compounds and plant extracts. J. Agric. Food Chem..

[168] Dorman H.J.D., Deans S.G. (2000). Antimicrobial agents from plants: Antibacterial activity of plant volatile oils. J. Appl. Microbiol..

[169] Pinho E., Henriques M., Soares G. (2014). Cyclodextrin/cellulose hydrogel with gallic acid to prevent wound infection. Cellulose.

[170] Ge W., Cao S., Shen F., Wang Y., Ren J., Wang X. (2019). Rapid self-healing, stretchable, moldable, antioxidant and antibacterial tannic acid-cellulose nanofibril composite hydrogels. Carbohydr. Polym..

[171] Kwon S.S., Kong B.J., Park S.N. (2015). Physicochemical properties of pH-sensitive hydrogels based on hydroxyethyl cellulose-hyaluronic acid and for applications as transdermal delivery systems for skin lesions. Eur. J. Pharm. Biopharm..

[172] Jiji S., Udhayakumar S., Rose C., Muralidharan C., Kadirvelu K. (2019). Thymol enriched bacterial cellulose hydrogel as effective material for third degree burn wound repair. Int. J. Biol. Macromol..

[173] Rivero-Buceta V., Aguilar M.R., Hernandez-Arriaga A.M., Blanco F.G., Rojas A., Tortajada M., Ramirez-Jimenez R.A., Vazquez-Lasa B., Prieto A. (2020). Anti-staphylococcal hydrogels based on bacterial cellulose and the antimicrobial biopolyester poly(3-hydroxy-acetylthioalkanoate-co-3-hydroxyalkanoate). Int. J. Biol. Macromol..

[174] Fan Y., Namata F., Erlandsson J., Zhang Y., Wagberg L., Malkoch M. (2020). Self-assembled polyester dendrimer/cellulose nanofibril hydrogels with extraordinary antibacterial activity. Pharmaceutics.

[175] Du S., Chen X., Chen X., Li S., Yuan G., Zhou T., Li J., Jia Y., Xiong D., Tan H. (2019). Covalent chitosan-cellulose hydrogels via schiff-base reaction containing macromolecular microgels for ph-sensitive drug delivery and wound dressing. Macromol. Chem. Phys..

[176] Guo X., Gao H., Zhang J., Zhang L., Shi X., Du Y. (2021). One-step electrochemically induced counterion exchange to construct free-standing carboxylated cellulose nanofiber/metal composite hydrogels. Carbohydr. Polym..

[177] Ali N.H., Amin M.C.I.M., Ng S.-F. (2019). Sodium carboxymethyl cellulose hydrogels containing reduced graphene oxide (rGO) as a functional antibiofilm wound dressing. J. Biomater. Sci. -Polym. Ed..

[178] Wang C., Niu H., Ma X., Hong H., Yuan Y., Liu C. (2019). Bioinspired, injectable, quaternized hydroxyethyl cellulose composite hydrogel coordinated by mesocellular silica foam for rapid, noncompressible hemostasis and wound healing. ACS Appl. Mater. Interfaces.

[179] Su C., Liu J., Yang Z., Jiang L., Liu X., Shao W. (2020). UV-mediated synthesis of carboxymethyl cellulose/poly-N-isopropylacrylamide composite hydrogels with triple stimuli-responsive swelling performances. Int. J. Biol. Macromol..

[180] Benitoufa S., Miled W., Trad M., Ben Slama R., Fayala F. (2020). Chitosan hydrogel-coated cellulosic fabric for medical end-use: Antibacterial properties, basic mechanical and comfort properties. Carbohydr. Polym..

[181] Wahid F., Hu X.H., Chu L.Q., Jia S.R., Xie Y.Y., Zhong C. (2019). Development of bacterial cellulose/chitosan based semi-interpenetrating hydrogels with improved mechanical and antibacterial properties. Int. J. Biol. Macromol..

[182] Youssef A.M., Hasanin M.S., El-Aziz M.E.A., Turky G.M. (2021). Conducting chitosan/hydroxylethyl cellulose/polyaniline bionanocomposites hydrogel based on graphene oxide doped with Ag-NPs. Int. J. Biol. Macromol..

[183] Noipitak P., Inphonlek S., Nillawong M., Sunintaboon P., Amornsakchai T. (2021). Chitosan/alginate composite porous hydrogels reinforced with PHEMA/PEI core-shell particles and pineapple-leaf cellulose fibers: Their physico-mechanical properties and ability to incorporate AgNP. J. Polym. Res..

[184] Khamrai M., Banerjee S.L., Paul S., Samanta S., Kundu P.P. (2019). Curcumin entrapped gelatin/ionically modified bacterial cellulose based self-healable hydrogel film: An eco-friendly sustainable synthesis method of wound healing patch. Int. J. Biol. Macromol..

[185] Anagha B., George D., Maheswari P.U., Begum K.M.M.S. (2019). Biomass derived antimicrobial hybrid cellulose hydrogel with green ZnO nanoparticles for curcumin delivery and its kinetic modelling. J. Polym. Environ..

[186] Dacrory S., Abou-Yousef H., Abouzeid R.E., Kamel S., Abdel-Aziz M.S., El-Badry M. (2018). Antimicrobial cellulosic hydrogel from olive oil industrial residue. Int. J. Biol. Macromol..

